# Generation and propagation of bursts of activity in the developing basal ganglia

**DOI:** 10.1093/cercor/bhad307

**Published:** 2023-08-23

**Authors:** Sebastian Klavinskis-Whiting, Sebastian Bitzenhofer, Ileana Hanganu-Opatz, Tommas Ellender

**Affiliations:** Department of Pharmacology, University of Oxford, Mansfield Rd, Oxford, OX13QT, United Kingdom; Department of Biomedical Sciences, Institute of Developmental Neurophysiology, Center for Molecular Neurobiology Hamburg (ZMNH), University Medical Center Hamburg-Eppendorf, 20246 Hamburg, Germany; Department of Biomedical Sciences, Institute of Developmental Neurophysiology, Center for Molecular Neurobiology Hamburg (ZMNH), University Medical Center Hamburg-Eppendorf, 20246 Hamburg, Germany; Department of Pharmacology, University of Oxford, Mansfield Rd, Oxford, OX13QT, United Kingdom; Department of Biomedical Sciences, University of Antwerp, Universiteitsplein 1, 2610 Wilrijk, Belgium

**Keywords:** development, network bursts, oscillations, spindle bursts, nested gamma spindle bursts, striatum, cortex, intralaminar thalamus

## Abstract

The neonatal brain is characterized by intermittent bursts of oscillatory activity interspersed by relative silence. Although well-characterized for many cortical areas, to what extent these propagate and interact with subcortical brain areas is largely unknown. Here, early network activity was recorded from the developing basal ganglia, including motor/somatosensory cortex, dorsal striatum, and intralaminar thalamus, during the first postnatal weeks in mice. An unsupervised detection and classification method revealed two main classes of bursting activity, namely spindle bursts and nested gamma spindle bursts, characterized by oscillatory activity at ~ 10 and ~ 30 Hz frequencies, respectively. These were reliably identified across all three brain regions and exhibited region-specific differences in their structural, spectral, and developmental characteristics. Bursts of the same type often co-occurred in different brain regions and coherence and cross-correlation analyses reveal dynamic developmental changes in their interactions. The strongest interactions were seen for cortex and striatum, from the first postnatal week onwards, and cortex appeared to drive burst events in subcortical regions. Together, these results provide the first detailed description of early network activity within the developing basal ganglia and suggest that cortex is one of the main drivers of activity in downstream nuclei during this postnatal period.

## Introduction

The developing brain is a hugely dynamical system, with marked changes in structural organization and functional connectivity occurring on both fast (seconds) and slow (days) timescales (Stiles and Jernigan 2010). It has been demonstrated that transient bursts of neural activity, which characterize the brain during this dynamic period, are critical for many developmental processes including neuronal migration and synaptic refinement (Katz and Shatz 1996; Heck et al. 2008; Bonetti and Surace 2010; Yasuda et al. 2011; Bando et al. 2016; Minocha et al. 2017; Suchkov et al. 2018; ). Disruption of these early patterns of activity can lead to aberrant neuronal circuit development and lasting cognitive impairments in animal models (Hartung et al. 2016b; Chini et al. 2020; Bitzenhofer et al. 2021; Chini and Hanganu-Opatz 2021) and likely contribute also to the emergence of a variety of neurodevelopmental and neurological disorders including schizophrenia, autism, and epilepsy, amongst others (Graybiel and Rauch 2000; Del Campo et al. 2011; Langen et al. 2011; McNaught and Mink 2011; Shepherd 2013; Albin 2018; Molnar et al. 2020; Chini and Hanganu-Opatz 2021; Iannone and De Marco Garcia 2021; Luhmann et al. 2022). Many studies of these patterns of activity have been performed in cortical structures (Hanganu et al. 2006; Brockmann et al. 2011; An et al. 2014; Cichon et al. 2014; Shen and Colonnese 2016) or related afferent structures such as thalamus (Weliky and Katz 1999; Minlebaev et al. 2011; Yang et al. 2013). In these brain regions, many different activity patterns have been described, which are typically classified based on their distinct properties (e.g. amplitude, duration) and spectral structure, and can either be spontaneously generated (Hanganu et al. 2006; Yang et al. 2013; Luhmann et al. 2016; Martini et al. 2021) or evoked by external sensory inputs (Khazipov et al. 2004; Milh et al. 2007; Yang et al. 2013; Gerasimova et al. 2014). These transient bursts of activity largely disappear at later stages of development and are replaced by longer duration oscillatory activity whose spectral components have themselves been related to diverse physiological and cognitive functions (Boraud et al. 2005; Colgin 2013; Khazipov et al. 2013).

How these transient patterns of early bursting activity impact or interact with many other downstream parts of the developing brain, including the basal ganglia, is largely unknown. The basal ganglia consist of a group of subcortical brain nuclei essential for the control of movement, as well as a variety of other functions in adulthood (Graybiel et al. 1994; Grillner et al. 2005; Yin and Knowlton 2006). As early patterns of activity can travel widely and coordinate patterns of activity amongst disparate brain regions (Ackman et al. 2012; Hartung et al. 2016a; Ahlbeck et al. 2018), it is highly likely that they also propagate through the basal ganglia. The striatum is the principal input nucleus of the basal ganglia that receives its main excitatory synaptic inputs from the cortex and thalamus (Buchwald et al. 1973; Smith et al. 2004; Kreitzer 2009; Doig et al. 2010; Ellender et al. 2013; Smith et al. 2014; Hunnicutt et al. 2016). Although the direct cortico-striatal inputs come from widespread cortical areas (Oh et al. 2014; Hunnicutt et al. 2016), the main thalamic inputs to the striatum are thought to arrive predominantly from the intralaminar nuclei of the thalamus (Macchi et al. 1984), which in rodents are divided in the rostral central lateral (CL) and the caudal parafascicular (Pf) nuclei (Berendse and Groenewegen 1990; Smith et al. 2009) and which are integrated in larger thalamocortical loops (Mandelbaum et al. 2019). Direct striatal input from primary sensory thalamic nuclei is thought to be minimal or non-existent to striatum, at least in adulthood (Alloway et al. 2017; Ponvert and Jaramillo 2019). The excitatory synaptic inputs to striatum become functional during the first postnatal weeks (Tepper et al. 1998; Nakamura et al. 2005; Dehorter et al. 2011; Peixoto et al. 2016; Krajeski et al. 2019) and innervate both the spiny projection neurons (SPNs) and interneurons of the striatum (Sadikot et al. 1992b; Castle et al. 2005; Lacey et al. 2007; Doig et al. 2010; Ellender et al. 2013; Smith et al. 2014; Assous et al. 2017; Johansson and Silberberg 2020). Similar to cortex, appropriate levels of neural activity has been shown to be necessary for the proper development of synapses and circuits in the early postnatal striatum (Kozorovitskiy et al. 2012; Peixoto et al. 2016; Peixoto et al. 2019) as well as survival of striatal neurons (Sreenivasan et al. 2022). Indeed, aberrant levels of activity during early development can cause permanent striatal dysfunction (Mowery et al. 2017; Vicente et al. 2020). Despite these important functions for early neural activity, very little is known about the diversity of activity patterns or their interactions amongst the basal ganglia nuclei.

Here, we employed multi-site in vivo silicon probes to record early network activity in young mice during the first postnatal weeks (postnatal day (P)5 to P15) from three key nodes in the basal ganglia network, including the motor/somatosensory cortex, the intralaminar thalamic nuclei, and the dorsal striatum. We focused on these three different regions to respectively capture the main excitatory afferent structures to the basal ganglia, as well as the main input nucleus of the basal ganglia. We applied an unbiased detection and classification method to these patterns of activity and explored to what extent these activity patterns propagated and interacted in amongst brain regions and distinct developmental time periods. We find that patterns of brief bursting activity in the developing basal ganglia are dominated by spindle (~10 Hz) and beta-low gamma (~15–30 Hz) frequency bands, corresponding to spindle bursts (SB) and nested gamma spindle bursts (NGB), respectively, which were found in all three key nodes. These exhibited differing characteristics and developmental profiles and interestingly further analysis suggested they mediate distinct directional interactions amongst basal ganglia nuclei during early postnatal development.

## Materials and methods

### Animals

All experiments were carried out on C57Bl/6 wildtype mice of both sexes with ad libitum access to food and water. Experiments were designed to use litter mates for extended developmental ranges within single experiments, as to control for effects of litter sizes and maternal care factors that could affect the degree of neuronal and circuit maturity. All mice were bred, individually ventilated cages (IVC) housed in a temperature-controlled animal facility (normal 12:12 h light/dark cycles) and used in accordance with the UK Animals (Scientific Procedures) Act (1986).

### Surgery and acute extracellular recording conditions

All experiments were conducted in accordance with the national laws and the UK guidelines for the use of animals in research and approved by the local ethical committee. Multi-site extracellular silicon probe recordings were performed unilaterally in the cortex, striatum, and thalamus of postnatal day 5 (P5) to P15 mice using experimental protocols as described previously (Hanganu et al. 2006; Brockmann et al. 2011). Coordinates were for cortex and striatum (0.5 mm anterior to bregma and 3.5 mm from the midline at a 30° angle and 1.5 mm depth) and thalamus (1.0–1.6 mm posterior to bregma and 0.7 mm from the midline and 2.7–3.1 mm depth and specifically P5–6; AP (anterior–posterior): −1.1/−1.2 mm ML (medial-lateral): 0.7 mm DV (dorso-ventral): 2.7 mm, P7–8; AP: −1.3/−1.4 mm, ML: 0.7 mm, DV: 2.8 mm, P9–10; AP: −1.5/−1.6 mm, ML: 0.7 mm, DV: 2.9 mm and P11–12; AP: −1.6/−1.7 mm, ML: 0.7 mm, DV: 3.1 mm) Briefly, urethane was injected intraperitoneally (1 mg/g body weight) prior to the surgery, and under isoflurane anesthesia, a plastic bar head mount was fixed with dental cement to the nasal and occipital bones. A craniotomy was performed above the recording locations, taking care not to pierce the dura mater, by drilling a hole of ~ 0.5–1 mm in diameter. The head was fixed into the stereotaxic apparatus (Stoelting) using the plastic bar head mount. During recordings, the body of the animals was surrounded by cotton and kept at a constant temperature of 37°C using a heating blanket. Multi-site electrodes (NeuroNexus, MI, USA) were inserted into the brain (A16, 5 mm length, cortex/striatum: 100 μm spacing and thalamus: 50 μm spacing). Silver wires were inserted into the cerebellum and served as ground and reference. After 30 min recovery, the recordings were started from silicon probes to obtain simultaneous recordings of field potential (FP) and multiple-unit activity (MUA) at different depths and locations. The electrodes were labeled with DiIC18(7) (1,1’-dioctadecyl-3,3,3′,3’-tetramethylindotricarbocyanine iodide) (DiR) (Invitrogen) to enable post-mortem reconstruction of the electrode tracks in histological sections.

### Impairment of cortical activity

Acute and reversible impairment of cortical activity was induced by injection of 300 nl of lidocaine (Sigma-Aldrich; 10% in 0.9% saline, pH 7.0 with NaOH, 100 nl/min) into upper layers of cortex. The lidocaine acted on a small tissue volume within an estimated radius of 300–400 μm (Tehovnik and Sommer 1997), and, consequently, the lidocaine-induced impairment of electrical activity is thought to be confined to cortex. Cortical injections consisted of two injections made at 0.5 mm depth superficial to recording locations (i.e. 0.5 mm anterior to bregma and 3.5 mm from the midline and 1.0–1.6 mm posterior to bregma and 0.7 mm from the midline).

### Data acquisition

Recordings lasted between 30 and 60 min. Data were acquired at a sampling rate of 30 kS/s using the Open Ephys acquisition system (Siegle et al. 2017). Silicon probes were connected to electrode adapter boards to 16-channel recording headstages (Intan Technologies) that were each connected to the Open Ephys acquisition board which was connected to a Dell Latitude 7370 laptop.

### Data analysis

Data were analyzed offline using custom written scripts in Igor Pro (Wavemetrics, RRID:SCR_000325) and Python. Analysis code is accessible at https://github.com/sebbkw/networkbursts2022. Data were filtered offline at 1–100 Hz for local field potential (LFP) analyses or 400–4000 Hz for MUA analyses using a third-order Butterworth filter. From each recording session, the data channel for analysis was selected out of three channels which best coresponded to the required anatomical location (i.e. cortex, dorsal striatum and intralaminar thalamus) and exhibited the lowest amount of noise by visual inspection. In addition, in three recording sessions, the presence of electrical noise artifacts was deemed too large, and these were not used for subsequent analysis.

#### Spike detection

Spikes were detected by applying a threshold set at 5 standard deviations below baseline MUA (Muthmann et al. 2015). Candidate spike waveforms were extracted by selecting the 30 time-samples (1 ms) surrounding the local minimum over each set of consecutive time points crossing the threshold. Spikes were rejected if the maximum point did not exceed 0 μV.

#### Burst detection

Two burst detection methods—envelope thresholding and root mean square (RMS) thresholding—were initially trialed (see Supplementary Fig. 1). For both methods, the LFP signal was first band-pass filtered between 4 and 100 Hz to remove low-frequency artifacts that could bias the detection of bursts. As no ground truth is available (i.e. there is no objective definition for which time periods constitute bursting events), the use of two independent methods provided an additional means of validating burst detection by considering whether the two methods produced comparable results. Overall, overlap was good for striatum (mean overlap: 84%, SD: 11%) and thalamus (mean overlap: 82%, SD: 14%), indicating that both successfully identified burst events with some method-specific variability. Ultimately, the RMS thresholding method was chosen for all subsequent analyses as it has been better validated in the literature and deemed more parsimonious insofar as it does not require any per-recording parameters to be adjusted by the experimenter. At around P12, a developmental switch was observed whereby bursting events were no longer discernible from continuous oscillatory activity. Accordingly, all subsequent analyses for bursting events were only performed using data from animals aged P5–12. The RMS thresholding method was adapted from Cichon and colleagues (Cichon et al. 2014). In brief, RMS values were first computed for each 200 ms window of the LFP signal before being binned to produce a distribution of RMS values. The RMS threshold was determined by fitting a Gaussian function to this RMS distribution, taking the threshold as μ + 3*σ*, where μ is the mean and σ is the standard deviation of the fitted Gaussian. Burst events were defined as those 200 ms time periods where the RMS exceeded the RMS threshold. At older ages (i.e. *P* > 10) where the proportion of bursting events relative to baseline periods increased, a naive curve-fitting procedure tended to overestimate the optimal threshold such that only the peaks of bursting events were detected. Therefore, and unlike for Cichon et al. (2014), the Gaussian mean was limited to a maximum RMS value of 50 μV during curve fitting. All consecutive 200 ms periods that exceeded the threshold were combined and defined as a single burst event. Several criteria were applied to further ensure the quality of detected bursts and to minimize the occurrence of false positives (i.e. periods of silence identified as burst events). Firstly, putative burst events were rejected if they were below 0.2 s or above 20 s in duration. Although exceedingly long (40–80 s) spindle-like bursts have been described in certain parts of cortex (Yang et al. 2013) no such bursts were observed in our recordings by visual inspection. Moreover, as most bursting activity in cortex in general has been shown to be relatively transient in the order of 1–10 s (Minlebaev et al. 2011; Khazipov et al. 2013; Gerasimova et al. 2014; Suchkov et al. 2018) we excluded bursts exceeding 20 s from analysis. The total percentage of rejected events was 1.3% for cortex, 2% for striatum, and 1.3% for thalamus. Secondly, bursting events were required to have at least five peaks above the mean RMS for that burst event to exclude non-specific increases in LFP amplitude not accompanied by oscillatory activity. Thirdly, motion artifacts were accounted for by rejecting all events with a mean RMS value exceeding 1,000 μV.

#### Unsupervised burst classification

Burst events were classified using a combined principal component analysis (PCA) and clustering approach. PCA was used as a feature extraction method to identify latent variables in the feature space which could then be used to cluster bursting events into different groupings. This data-driven approach was both unsupervised and unbiased as compared with manual classification methods, as bursts were classified using those axes that explained maximal variance in the data rather than relying on predefined criteria. Each burst event was used to produce a feature vector on which the PCA procedure was performed. Nine features were included for the analysis: duration, negative peak, maximum RMS, flatness, maximum slope, inter-trough interval (ITI), relative theta-alpha power, relative beta-low gamma power, and spike rate. These distinct features were selected to capture a variety of aspects of each bursting event including their structural and spectral characteristics as validated in previous work (Cichon et al. 2014; Hartung et al. 2016a, 2016b). For all features other than relative theta-alpha power and relative beta-low gamma power, burst events were band-pass filtered at 4–100 Hz. The duration was computed as the difference between the start and end times of the detected burst event. The negative peak was defined as the minimum deflection in the LFP signal. The maximum RMS was defined as the maximum RMS values computed across 200 ms chunked time windows. Flatness was defined as min(RMS)*/*max(RMS) out of all 200 ms period RMS values across the burst event. Slope was taken as the instantaneous difference in voltage between consecutive time points, for which the burst event was downsampled to 500 S/s to ensure that inter-sample time points were large enough to produce sufficient variation in slope across burst events. The ITI was defined as the mean time interval between all troughs in the LFP signal, where local minima were included as troughs only if their prominence exceeded half the RMS of the burst event. Spectral measures were taken as the power in the theta-alpha (4–16 Hz) and beta-low gamma (16–40 Hz) bands relative to the total power of the normalized power spectra in the frequency range 1–50 Hz. Spike rate was defined as the number of spike events occurring per second over the course of the burst event. Prior to running the PCA, all features were normalized. Bursts were then clustered using the first three components extracted by the PCA. Clustering was performed using the fuzzy c-means clustering algorithm, where—in contrast to hard clustering algorithms—each point is assigned a relative probability for its inclusion to each cluster (Ross 2010). By virtue of this “fuzzy” approach, clustering could make use of a threshold whereby points of low confidence for inclusion to any cluster were labeled as unclassified. For clustering in this study, burst events were labeled as unclassified if the maximum coefficient did not exceed 0.6, indicating less than 60% probability of the burst belonging to either of the two candidate clusters. The clustering threshold was set as a compromise between ensuring that low-confidence bursts were not included in either cluster and that excessive burst events were not unduly discarded from further analysis. The optimal number of clusters was determined by measuring the fuzzy partition coefficient (FPC) as a function of cluster number, where the FPC denotes the quality of the resulting partition with an optimal value of 1. Excluding the trivial case where *n* = 1, the optimal number of clusters was determined as *n* = 2 for both striatal (FPC = 0*.*75) and thalamic (FPC = 0*.*73) burst events (Supplementary Fig. 3).

#### Time-frequency analysis

LFP power spectra were estimated using the multi-taper method via the “pmtm” function in the Spectrum toolbox (Cokelaer and Hasch 2017). For all power spectral density (PSD) analyses, 1 s windows were advanced by 0.1 s with a half-bandwidth parameter of 3 using the first five Slepian sequences. To account for non-specific spectral properties during bursting events, all PSDs were normalized (*P/P*_0_) according to the PSD measured during baseline non-bursting time periods (Cichon et al. 2014; Gretenkord et al. 2019). The baseline PSD was calculated as the mean PSD across all periods in each recording not included as bursting events, which exceeded 1 s in duration. Spectrograms were produced purely for display purposes using the SciPy “spectrogram” function in the signal toolbox, using a time window of 0.5 s with a 99% overlap across segments.

#### Coherence analyses

Bursts were considered to be co-occurring if their onset overlapped by less than 0.5 s. The frequency of co-occurring burst types (SB/SB, NGB/NGB, NGB/SB) was normalized by the total number of bursting events in each class to control for their absolute incidence. Cross-spectral coherence between co-occurring burst events was then computed using Welch’s method using the “coherence” function in the SciPy signal toolbox (Virtanen et al. 2020). Cross-spectral coherence was computed with a time window of 0.5 and 0 s of overlap across segments. Significance was determined by shuffling all pairs of burst events 1,000 times and computing the mean coherence at each iteration to produce a null distribution (see Supplementary Fig. 8). The significance threshold was calculated by computing the 95th percentile of the resulting null distribution. Co-occurring events with a mean cross-spectral coherence exceeding 0.8 were rejected, as these events of high synchrony are likely to have resulted from artifacts such as movement (Hartung et al. 2016a, 2016b). Imaginary coherence was computed as the absolute of the imaginary component of the normalized cross-spectrum using Welch’s with a time window of 0.5 and 0 s of overlap across segments (Gretenkord et al. 2019). Significance was determined by shuffling all pairs of burst events 1,000 times and computing the mean coherence at each iteration to produce a null distribution. Spike-field coherence was computed as previously described (Soteropoulos and Baker 2006). Spike trains were binned in 2 ms time-segments to produce a continuous waveform, with LFP signals (4–80 Hz bandpass filtered) similarly down sampled to match the spike-train sampling rates. Cross-spectral coherence between these two signals was then computed as the real component of the normalized cross-spectrum as described above. The significance of synchrony was assessed by constructing a null distribution across 1,000 iterations where spike timings were randomly shuffled.

#### Cross-correlation analyses

Causal interactions between brain regions were assessed by means of a cross-correlation analysis (Adhikari et al. 2010; Hartung et al. 2016a). First, LFP signals were band-pass filtered around the frequencies of interest—here, the 4–16 and 16–40 Hz frequency windows around which spectral power was greatest for SB and NGB bursts, respectively. The LFP signal was then pre-whitened to remove autocorrelations which can produce spurious correlations between input signals (El-Gohary and McNames 2007). More specifically, pre-whitening was achieved by fitting an ARIMA model to the LFP signal before computing the normalized cross-correlation on the resulting residuals of the fitted model (Merchant et al. 2014). The lag was then taken as the time lag between +50 and − 50 ms, which produced the maximal cross-correlation. For cortical-striatal bursts, a positive lag indicates the striatum leading the cortex and a negative lag the cortex leading the striatum. For thalamic-striatal bursts, a positive lag indicates the striatum leading the thalamus and a negative lag the thalamus leading the striatum. Finally, for cortical-thalamic bursts, a positive lag indicates the thalamus leading the cortex and a negative lag the cortex leading the thalamus. Shorter lags of less than 20 ms were considered putative monosynaptic interactions, whereas longer lags were indicative of possible polysynaptic interactions (Hartung et al. 2016a).

#### Spike train cross-correlation/lag analysis

Spike times from the co-occurring burst events were first extracted from the filtered MUA signal as described above, after which spike trains were convolved with a Gaussian kernel with a standard deviation of 2 ms to produce a continuous signal. The normalized cross-correlations of these two signals were then computed and the peak lag taken between +/− 10 ms. Significance was assessed using a one-sample *t*-test across the peak lag from each burst pair.

#### Spike analysis lidocaine experiments

Spiking activity was extracted as described above with activity taken from 5 min onwards from control recordings and between 5 and 25 min into lidocaine recordings. The smaller time window for lidocaine recordings ensured that inactivation of the target structure was maximal, ignoring later time points where the effect of lidocaine was likely to be minimal or reduced. Finally, the spike rate for each brain region was averaged across three channels, centred on the target channel.

### Histological analyses

Following recordings, the mice were culled, and brains immediately transferred to ice-cold 4% paraformaldehyde in 0.1 M PBS and fixed for three or more days. Brains were then sectioned on a Leica VTS1000 vibratome and coronal sections (40 μm) collected. All sections were mounted in Vectashield (Vector Laboratories, Cat. H-1000) and DiR recording tracts were immediately analyzed. Images were captured with a Leica epifluorescence microscope using HCImage software (Hamamatsu). Images were processed and analyzed in ImageJ and Adobe Photoshop and Illustrator.

### Statistics

Statistics were computed using data binned into two-day age groups (namely P5–6, 7–8, 9–10, 11–12). Recordings were made from a total of 23 animals and in the majority of cases consisted of simultaneous recordings from cortex/striatum and thalamus for (P5–6; *n* = 4 mice cortex/striatum and *n* = 3 mice also including thalamus, P7–8; *n* = 3 and *n* = 2, P9–10; *n* = 8 and *n* = 5, P11–12; both *n* = 4, P13–15; both *n* = 4). In addition, recordings were made simultaneously from cortex/striatum and thalamus from a total of 11 animals for lidocaine experiments which were analyzed as a single group (P5–6; *n* = 1, P7–8; *n* = 3, P9–10; *n* = 4, P11–12; *n* = 3). Age-dependent effects were assessed by means of two-way ANOVA with factors of age group and either burst-type (SB and NGB) or brain region (cortex, striatum, and thalamus). To assess the dependence of burst features on brain area, we compared the same burst type across different brain areas using a non-parametric approach to multivariate analysis of variance (Anderson 2001). Although formally equivalent to a *t*-test where the number of groups *K* = 2, this multivariate approach avoided the need for many multiple comparisons across groups and features. As for the univariate case, the *F* statistic was computed as follows:


$$ F=\frac{S{S}_A}{S{S}_W} $$

*SS_A_* is the “amongst” or between group sum-of-squares, defined as


$$ S{S}_A=\frac{\sum_{i=1}^K{n}_i{\left\Vert \overline{Y}-\overline{Y_i}\right\Vert}^2}{K-1} $$

where *K* is the total number of groups (here, 2), *n_i_* is the number of observations in group *i*, and $\left\Vert \overline{Y}-\overline{Y_i}\right\Vert$ is the Euclidean distance between the overall mean feature vector $\overline{Y}$ and the group mean feature vector $\overline{Y_i}$. *SS_W_* is the within group sum-of-squares, defined as


$$ S{S}_W=\frac{\sum_{i=1}^K{\sum}_{j=1}^{n_i}{\left\Vert \overline{Y_i}-{Y}_{ij}\right\Vert}^2}{N-K} $$

where $\left\Vert \overline{Y_i}-{Y}_{ij}\right\Vert$ is the Euclidean distance between individual feature vector *Y_ij_* and group mean feature vector $\overline{Y_i}$, and *N* is the total number of feature vectors across all groups. To assess the significance of the *F*-statistic, groups were shuffled 10*,*000 times, computing the test statistic each time to produce a null distribution against which the significance of the true *F*-statistic could be determined. Further statistical details of experiments can be found in the respective Results sections and Figure legends. For all statistical tests, the significance level was set at α = 0.05, with p-values corrected for multiple comparisons using Bonferroni correction where relevant. All error bars are given as standard error of the mean (SEM) except when stated otherwise. Absolute *P*-values are given for all values except for permutation tests where the lower bound was based on the number of permutations (e.g. *P* < 0.0001 for 10,000 permutations). Significance is indicated in figures as ^*^P < 0.05, ^*^^*^P < 0.01, ^*^^*^^*^P < 0.001.

## Results

### Multi-channel recordings of early neural activity in the developing basal ganglia

**Fig. 1:**
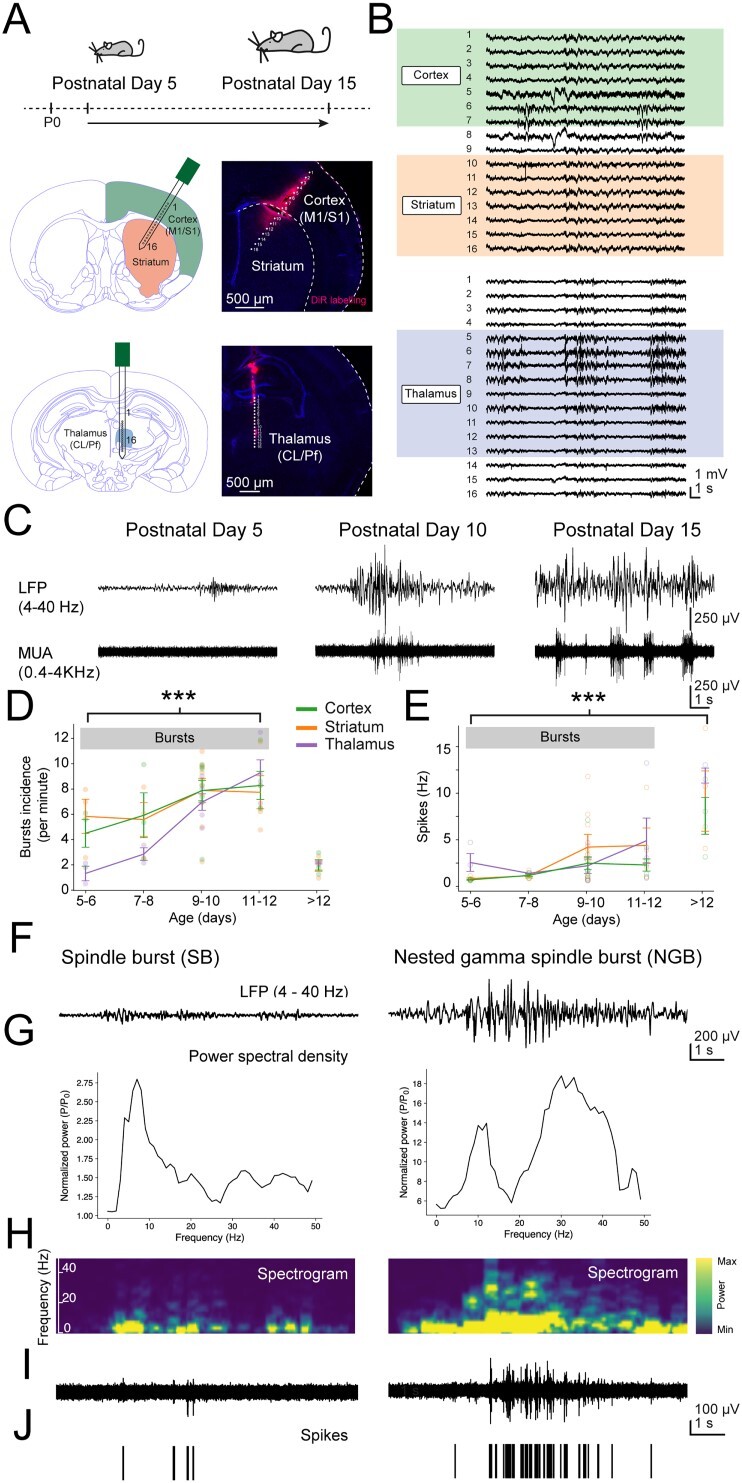
Bursts of activity in the basal ganglia increase during early postnatal development, which resemble spindle and gamma bursts. (A) Recordings were made from the cortex (M1/S1 border), dorsal striatum, and intralaminar thalamus of mice between postnatal day (P)5 and P15 using a 16-channel silicon probe inserted at a 30° angle to simultaneously record from cortex and striatum and an additional 16-channel probe inserted vertically to record from the intralaminar thalamus, including the CL and Pf nuclei. (B) Example field potential recordings made from the channels located in cortex and striatum (top) and thalamus (bottom) at P12. Note the heterogeneity in the amplitude, duration, and localization of bursts. (C) Example striatal field potential recordings after 4–40 Hz band-pass filtering to resolve bursts in the LFP (top) and 0.4–4KHz band-pass filtering to resolve spiking or MUA (bottom). Note the general transition from small and infrequent bursts interspersed by periods of silence to continuous activity patterns in the LFP concomitant with an overall increase in spiking activity across early postnatal development. (D) Overall burst incidence modestly increased during early postnatal development. During later stages, bursts were replaced with continuous oscillatory activity as observed in all three brain regions. (E) MUA increased in all three brain regions and was most pronounced during later stages of postnatal development. (F) Example striatal SB and NGB in 4–40 Hz bandpass filtered LFP signal (top) as recorded at postnatal day (P)8 and P10, respectively. (G) PSD plot of relative spectral power within bursts, normalized to baseline power. Note the prominent peaks at ~ 5–10 Hz in both bursts and the additional peak at ~ 20–40 Hz in the NGB. (H) Spectrogram of the corresponding LFP signal. Note the presence of beta-gamma band (~20–40 Hz) activity during the mid-phase of the NGB. (I) 0.4–4 kHz MUA filtered activity. (J) Raster plot of spikes extracted from MUA. Note that NGB are characterized by increased spiking at higher frequencies as compared with SB. Individual bursts and spiking events are averaged per animal, which are plotted according to age in (D) and (E).

The basal ganglia are a complex interconnected network of subcortical nuclei of which the striatum is the main input nucleus receiving excitatory synaptic inputs from both extended regions of cortex and diverse thalamic nuclei (Hunnicutt et al. 2016). The developing brain generates bursts of activity but how these propagate and interact between different basal ganglia regions is largely unknown. Here, recordings of early neural activity were made using two 16-channel silicon probes placed in the cortex at the motor and somatosensory border, the dorsal striatum as well as the intralaminar thalamus, including both the CL and Pf nuclei, in young postnatal mice ranging in age from postnatal day (P)5 to P15 (Fig. 1A). Recordings were made from a total of 23 animals under urethane anesthesia, which is thought to minimally interfere with naturalistic brain activity and its spectral characteristics (Chini et al. 2019). These recordings revealed extended periods of quiescence interspersed with transient oscillatory events as detected from subsets of channels and reflecting neuronal activity within specific brain regions (Fig. 1B). These recordings were analyzed using unbiased burst detection algorithms (see Methods, Supplementary Fig. 1 and Cichon et al. 2014). Initial analysis of recordings suggested that neural activity, as assessed at the level of LFPs and spikes or MUA, exhibited gradual changes during postnatal development. At P5, LFP activity was mostly characterized by electrical silence, interspersed with small deflections likely reflecting the first beginnings of synchronized neural activity (Fig. 1C). At later stages, bursting events became more apparent, as larger but still transient increases in synchronous activity, as seen in both the LFP and MUA signals and interspersed by periods of silence. Finally, by the start of the third postnatal week the periods of silence became infrequent, and the brain state became predominantly active, indicating a more mature state akin to that found also in the adult brain (Fig. 1C, D). The overall developmental trajectory appeared similar across cortex, striatum, and thalamus, indicating a gradual progression from a relatively quiet brain state toward one that generated intermittent bursts of activity before the onset of a continuous active state. Indeed, the incidence of bursts in all three brain regions increased significantly across early development (*F*(4, 42) = 12.1, *P* = 0.000001), before declining at later stages of development (>P12) when all areas began to exhibit continuous oscillatory activity (Fig. 1C, D). Initial analysis of data filtered to reveal spikes (MUA, 0.4–4 kHz) demonstrated an overall increase in MUA in all three brain regions (*F*(4,45) = 8.83, *P* = 0.000024, Fig. 1C, E). The maturation of neuronal activity within these different brain regions was not only evident in overall increases in bursting incidence and spike frequency but was also reflected in other measured parameters such as burst amplitude and the time occupied by burst events (Supplementary Fig. 2). All further analysis was performed on recordings up to P12 and was focused on the intermittent bursting activity only.

### Unbiased classification and clustering of network bursts in cortical recordings

**Fig. 2:**
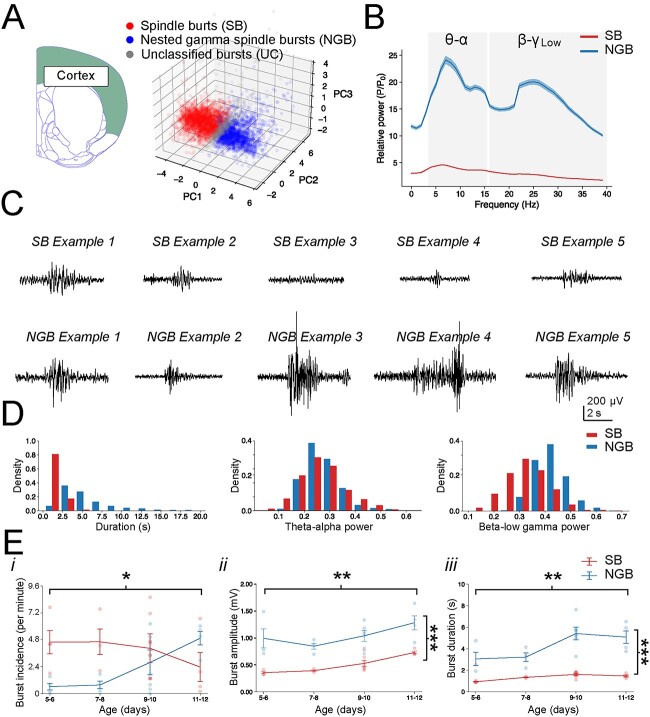
Unbiased detection and clustering of cortical events confirms the existence of two classes of bursts. (A) Scatter plot of the first three PCs used for clustering of cortical events into SB (a total of 2,237 events, red) and NGB (1,657 events, blue). Unclassified bursts (UC, 431 events) are denoted in gray. (B) PSD plot of the mean normalized power across SB and NGB events demonstrate that SB events exhibit prominent power in the spindle frequency range (theta-alpha (θ-α), 4–16 Hz), whereas NGB exhibit prominent power in the beta-low gamma frequency (β-γ_Low_, 16–40 Hz), which are nested within lower frequency spindle-band activity. (C) Example of recorded cortical SB and NGB events after automated detection and clustering. Note the mostly larger amplitude and longer duration of NGB events. (D) Histogram of the distribution of several key features across cortical SB and NGB events, including their duration, theta-alpha (θ-α) power, and beta-low gamma (β-γ_Low_) power. Note the consistently longer duration of NGB events and their greater beta-low gamma (β-γ_Low_) power. (E) Graphs depicting the developmental changes in the incidence (i), amplitude (ii), and duration (iii) of cortical SB and NGB events. Note the decrease in SB incidence and concomitant increase in NGB incidence during development, as well as an overall increase in the amplitude and duration of all events with the latter most pronounced for NGB events during later stages of development. Individual bursts are pooled across all animals and ages in (A), (B), (D) and averaged per animal, which are plotted according to age in (E).

Initial inspection of bursting events suggested that they fell into roughly two distinct classes based on the dominant frequency of their oscillations and these could often be observed in recordings from all three brain regions. One class of events was reminiscent of SB (Fig. 1F), due to their prominent power in the spindle frequency range (8–30 Hz) (Hanganu et al. 2006; Yang et al. 2016) (Fig. 1G, H), whereas the second class of events was reminiscent of NGB (Fig. 1F), due to the appearance of additional higher frequency beta-low gamma oscillations (20–40 Hz) (Fig. 1G, H), as previously observed, although occasionally referred to by different names, in prefrontal and prelimbic cortex and other regions (Brockmann et al. 2011; Khazipov et al. 2013; Yang et al. 2013; Cichon et al. 2014; Hartung et al. 2016a) (Fig. 1G, H). In addition to these spectral characteristics, the NGB often appeared longer in duration and exhibited increased MUA as compared with SB (Fig. 1I, J). Due to the similarities between SB and NGB events, e.g. both featuring elevated power at spindle frequencies and co-occurring at similar developmental time points, as well as to avoid risk of experimenter bias, e.g. toward known patterns of bursting activity based on the existing literature, all detected burst events were categorized quantitatively using a combined PCA and clustering approach (see Methods). In brief, each detected bursting event was used to produce a feature vector incorporating several parameters including burst duration, power in the theta-alpha and beta-low gamma frequency range and spike rate, amongst others. By applying PCA, these features could be mapped to a lower dimensional space and then segmented via a clustering algorithm to identify different classes of bursting events. This approach was first validated on the cortical recordings made at border of the motor and somatosensory cortex to investigate whether it allowed for detection of similar events as previously described in the literature (Khazipov et al. 2004; Hanganu et al. 2006; Yang et al. 2009; Brockmann et al. 2011; Yang et al. 2013; An et al. 2014; Cichon et al. 2014; Shen and Colonnese 2016) (Supplementary Fig. 3A). The clustering of detected events revealed two main classes of burst activity in the cortical recordings (Fig. 2A).

As these exhibited many characteristics similar to SB and gamma oscillations/bursts previously described in other cortical regions (Hanganu et al. 2006; Yang et al. 2009; Cichon et al. 2014; Yang et al. 2016; Hartung et al. 2016a), we refer to these from now on as SB and NGB. Indeed, the detected and clustered events included similar dominant frequency components (Fig. 2B, C) with a peak for SB within the 4–16 Hz or theta-alpha (θ-α) frequency band, and for NGB an additional faster frequency peak in the 16–40 Hz or beta-low gamma (β-γ_Low_) frequency band (Fig. 2D and Supplementary Tables 1 and 4). The NGB were mostly nested within an envelope of SB frequency oscillations and were reminiscent of those described in the prefrontal cortex where they are referred to as NG (Brockmann et al. 2011). Not only the dominant frequency components but also their developmental trajectory were similar to those previously described (Hanganu et al. 2006; Brockmann et al. 2011; An et al. 2014; Cichon et al. 2014; Shen and Colonnese 2016), including a progressive decrease in the incidence of SB and an increase in NGB events (SB: 4.6 ± 2.1 to 2.4 ± 2.6 bursts/minute and NGB: 0.6 ± 0.6 to 4.9 ± 1.2 bursts/minute, *F*(3, 30) = 3.35, *P* = 0.0318) with the latter exhibiting a consistently larger amplitude (SB: 502 ± 57.8 and NGB: 1,040 ± 231 μV, *F*(1, 28) = 66.0, *P* = 7.59e-09) and longer duration (SB: 1.34 ± 0.14 and NGB: 4.19 ± 1.09 s, *F*(1, 28) = 109, *P* = 3.73e-11, Fig. 2E) during development. These and other measured parameters (Supplementary Fig. 7A) suggest that the cortical bursts detected and classified here exhibit all the hallmarks of those previously described (Hanganu et al. 2006; Brockmann et al. 2011; An et al. 2014; Cichon et al. 2014; Shen and Colonnese 2016) and suggests this approach of detection and unbiased classification could be effective in detection of burst events in striatal and thalamic regions also.

### Classification of burst events in striatum and thalamus

**Fig. 3:**
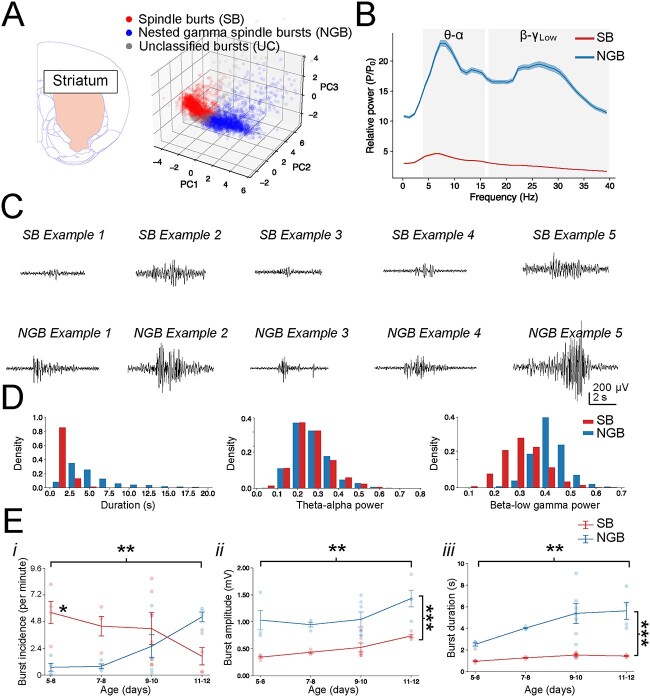
Striatal bursts cluster into two classes which exhibit properties consistent with SB and NGB events. (A) Scatter plot of the first three PCs used for clustering of striatal bursts into SB (a total of 2,242 clustered events, red) and NGB (1,633 events, blue). Unclassified bursts (UC, 437 events) are denoted by gray. (B) PSD of the mean normalized power across striatal NGB and SB events reveals that both events exhibit a prominent peak at theta-alpha frequency (θ-α) and that NGB events contain an additional peak at beta-low gamma frequency (β-γ_Low_). (C) Example SB and NGB events in striatum. Note the mostly larger amplitude of NGB events. (D) Histogram of the distributions of several key features across striatal SB and NGB events. (E) Graphs depicting the developmental changes in the striatal burst incidence (i), amplitude (ii), and duration (iii). Note the initial higher incidence and subsequent decrease of SB events (1*,*30) = 4*.*75*, P* = 0*.*0373) and concomitant increase in NGB incidence, as well as an overall increase in the amplitude (*F*(3*,*29) = 4.73*, P* = 0*.*00831) and duration (*F*(3*,*29) = 3*.*93*, P =* 0*.*0181) of events with the increase in duration most pronounced for NGB events. Individual bursts are pooled across all animals and ages in (A), (B), (D) and averaged per animal, which are plotted according to age in (E).

**Fig. 4:**
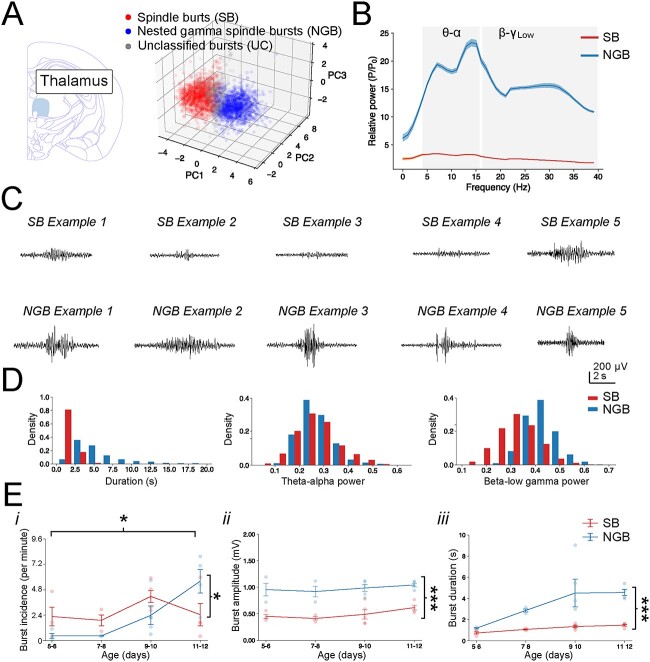
Thalamic bursts cluster into two classes exhibiting properties consistent with SB and NGB events. (A) Scatter plot of the first three PCs used for clustering of thalamic bursts into SB (1384 events, red), NGB (1335 events, blue) and UC bursts (409 events, gray). (B) PSD of the mean normalized power across SB and NGB thalamic events. (C) Examples of SB and NGB events in thalamus. (D) Histogram of the distributions of several key features across thalamic NGB and SB events. (E) Developmental changes in the burst incidence (i), amplitude (ii), and duration (iii). Thalamus does not exhibit as rapid a decline in SB events but note the comparable increase in thalamic NGB events across development. Similarly, the NGB events in thalamus exhibit consistently larger amplitudes and longer durations. Individual bursts are pooled across all animals and ages in (A), (B), (D) and averaged per animal, which are plotted according to age in E.

The burst detection and clustering method was next used on recordings from dorsal striatum (Fig. 3A, Supplementary Fig. 3B) and intralaminar thalamus (Fig. 4A, Supplementary Fig. 3C). Interestingly, the detected and clustered events in both striatum and thalamus exhibited many similar properties as those seen in cortex. It was therefore opted to refer to these clustered striatal and thalamic events as SB and NGB events as well. This decision was guided by several observations. First, the relative oscillatory power of striatal and thalamic SB and NGB events differed in that SB events had an overall lower oscillatory power as compared with NGB, similar to those in cortex (see e.g. Fig. 2B). Secondly, the mean PSD across SB and NGB events in both striatum and thalamus both exhibited a peak at the spindle frequency centred at 4–16 Hz (theta-alpha, θ-α) and NGB events exhibited an additional secondary peak centred at the 16–40 Hz (beta-low gamma, β-γ_Low_) range (Figs 3B, 4B and Supplementary Tables 2, 3 and 5, 6).

Thirdly, NGB events in both striatum and thalamus also were significantly longer in duration and had a greater spike rate than SB events (Figs. 3B, 4B and Supplementary Tables 2, 3 and 5, 6). Fourthly, the developmental trajectory of their properties mirrored that of cortex, with NGB incidence increasing with age at the cost of SB incidence (striatum: *F*(3*,*30) = 5*.*83*, P* = 0*.*00290 and thalamus: *F*(3*,*20) = 3*.*50*, P* = 0*.*0345), as well as the consistently greater amplitude (striatum: *F*(1*,*29) = 55*.*0*, P* = 3.59e-08 and thalamus: *F*(1*,*19) = 57*.*77*, P* = 3.56e-07) and duration (striatum: *F*(3*,*29) = 3*.*93*, P =* 0*.*0181 and thalamus: *F*(1*,*19) = 20*.*96*, P* = 0.000205, Figs 3E and 4E) of NGB events. In addition, many other developmental changes in their spectral properties also mirrored that seen in cortex (Supplementary Fig. 7B, C). Despite the many similarities amongst SB and NGB events in the brain regions studied, some subtle differences were observed. To explore this further, an initial multivariate extension of the *F*-ratio test was performed to determine whether the overall distributions of features across each class of burst events between the different brain regions differed (see Methods and Supplementary Fig. 5). This confirmed that SB and NGB events did exhibit some significant region-specific differences, with for example the distribution of features of SB events differing significantly between cortex and thalamus (*F*(1, 3619) = 30.2, adjusted *P* < 0.001) and cortex and striatum (*F*(1, 4477) = 53.8, adjusted *P* < 0.001; Supplementary Fig. 5A, C, E) and for NGB events differing significantly between cortex and thalamus (*F*(1, 2990) = 30.8, adjusted *P* < 0.001) and between striatum and thalamus (*F*(1,2966) = 22.8, adjusted *P* < 0.001; Supplementary Fig. 5B, D, F).

These observations likely result from a variety of subtle differences in burst properties. For example, for striatal NGB events the peak in the theta-alpha (θ-α) range was centred around ~ 5 Hz with significantly greater power in the 4–12 Hz range over the 12–20 Hz range (Welch’s *t*(2983) = 4.91, *P* = 9.60e-07), whereas for thalamic NGB events, the peak frequency was centred around ~ 12 Hz with significantly greater power in the 12–20 Hz range over the 4–12 Hz range (Welch’s *t*(2517) = 6.32, *P* = 3.03e-10; Figs 3B and 4B). Likewise, for striatal NGB events, a high spike rate was found during NGB events (9.75 ± 0.34 Hz) whereas, in thalamus, this was significantly lower (4.86 ± 0.15 Hz), and here the highest spike rate was in fact seen during SB events (6.51 ± 0.24 Hz, Supplementary Fig. 6 and Supplementary Tables 4, 5, and 6). However, in contrast, the equivalent F-ratios determining the distributions of features between SB and NGB events within single brain areas were several orders larger (cortex: *F*(1, 3892) = 8461, adjusted *P* < 0.001; striatum: *F*(1, 3873) = 8510, adjusted *P* < 0.001; thalamus: *F*(1, 2717) = 5464, adjusted *P* < 0.001; Supplementary Fig. 5G–I). Thus, even where variation exceeded significance between certain brain regions for the same class of events (Supplementary Tables 7–12), these differences were considerably smaller compared with variation between different classes of burst events within one brain region with effect sizes mostly small (range = 0.10–1.05).

Taken together, these results suggest that early network activity in the striatum and thalamus is dominated by the same two distinct classes of network bursts as seen in cortex, namely SB and NGB. In addition, they suggest that the properties of these events are mostly conserved between brain regions but exhibit subtle differences and likely reflecting the cellular environments of their generation and recording. Finally, in all three brain regions, a number of events had properties that did not fit in either category sufficiently, i.e. they often had characteristics in between those of SB and NGB events, and were denoted unclassified (UC, Supplementary Fig. 4) and not taken further in the analysis.

### Functional interactions amongst brain regions during bursting activity

**Fig. 5:**
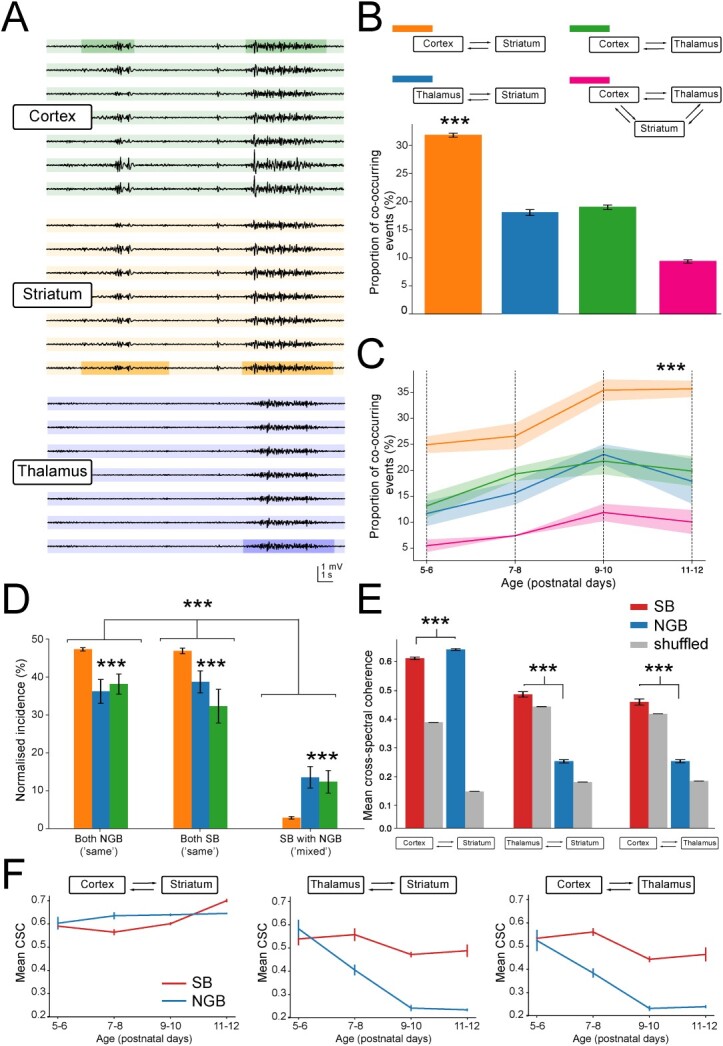
Co-occurring burst events are often of the same type and most pronounced between cortex and striatum. (A) The presence of co-occurring events was determined in the cortical, striatal, and thalamic regions recordings based on their onset. Note the co-occurring events in cortex and striatum (left) and in all three regions (right) in this example trace. Dark colored segments are specific traces and events that were used for analysis. (B) Co-occurring burst events are significantly more likely between cortex and striatum than between other brain regions. Only a small number of events co-occurred amongst all three brain regions (*M* = 0.09, SD = 0.04, all adjusted *P* ≤ 0.0134). (C) The proportion of co-occurring burst events significantly increases with postnatal age in all brain regions. Note that this is most pronounced for cortex-striatum between postnatal days 7–8 and 9–10. (D) Normalized incidence of NGB-NGB, SB-SB (both “same”), and NGB-SB (“mixed”) events amongst different brain regions. “Same” events are more likely than “mixed” events, implying that communication across brain areas may be mediated by the same rather than different burst types. No significant difference was seen in the normalized incidence of NGB/NGB versus SB/SB events (*t*(110) = 0.54*, P* = 0*.*593*, d* = 0*.*10). (E) The mean CSC is larger for NGB than SB for corticostriatal interactions. In contrast, this is larger for SB bursts event in both thalamostriatal and corticothalamic interactions. (F) Mean CSC across developmental time remained constant for all corticostriatal events but significantly declined for NGB events in thalamostriatal and corticothalamic interactions. Co-occurring bursts are pooled across all animals and ages in (B), (C), (D), (E) and plotted according to age in (F).

Having defined the different classes of bursts in the three brain regions we next determined how these bursts interacted with each other (Fig. 5A). We first investigated which pairs of brain regions had the most frequent co-occurring events, by taking the proportion of events whose onset overlapped out of the total number of burst events across the paired regions. This revealed that all brain regions exhibited events that co-occurred, but that the overall incidence of co-occurring events was greatest (~30% of all events) between cortex and striatum (all adjusted *P* < 0.001, Fig. 5B). In addition, there were many events that appear to be restricted to just one brain region only. This co-occurrence of events tended to increase during development for all combinations of brain regions (*F*(3, 41) = 8.50, *P* = 0.000164, Fig. 5C). Investigating whether co-occurring bursting events in general were more likely to be of the “same” class or different class (“mixed”) of burst event revealed there was a significant effect of burst type on the normalized incidence (*F*(2, 163) = 389, *P* = 9.59e-63), where the normalized incidence of co-occurring NGB/NGB events (*M* = 0*.*42*,* SD = 0*.*08) was significantly greater than co-occurring “mixed” NGB/SB or SB/NGB events (*M* = 0.07*,* SD = 0*.*08; *t*(109) = 22.9*, P* = 1.36e-43*, d* = 4.36), as was the normalized incidence of co-occurring SB/SB events (*M* = 0*.*42*,* SD = 0*.*11) events over mixed events (*t*(109) = 19.4*, P =* 4.88e-37*, d* = 3.62, Fig. 5D). Investigating this separately for brain region pairs revealed this appeared to be the case for all brain region pairs, although co-occurring bursts did differ more often in class (“mixed”) amongst cortex-thalamus and thalamus-striatum then in cortex-striatum (*F*(4, 163) = 17.3, *P* = 7.29e-12, Fig. 5D).

Having identified that bursts of the same type are more likely to co-occur in different brain regions next the synchrony between co-occurring bursts was quantified using cross-spectral coherence (CSC) analysis across pairs of SB and NGB events (Fig. 5E). Firstly, the mean CSC was significantly greater for both SB and NGB pairs compared with randomly shuffled data (*P* < 0.001, Supplementary Fig. 8 and Methods) indicating that observed coherence is above that expected by chance. Secondly, further analysis revealed that the mean CSC in cortex-striatum was greatest for both SB (*M* = 0.61, SD = 0.11) and NGB events (*M* = 0.64, SD = 0.09), as compared with other brain region pairs (SB and NGB, all adjusted *P* ≤ 2.02e-24; Fig. 5E). Thirdly, that within cortex-striatum the coherence amongst co-occurring events was greatest for NGB events (*t*(1219) = 5.87, *P* = 5.54e-09, Fig. 5E). In contrast, for thalamus-striatum events, coherence was significantly greater for SB (*M* = 0.48, SD = 0.11) over NGB events (*M* = 0.24, SD = 0.12; *t*(222) = −20.7, *P* = 7.95e-54), as was also the case for cortex-thalamus (SB: *M* = 0.45, SD = 0.13 and NGB: *M* = 0.24, SD = 0.11; *t*(218) = −17.7, *P* = 5.12e-44, Fig. 5E). To investigate whether certain frequency domains within co-occurring bursts of the same type exhibited greater coherence than others the coherence spectra within the 4–40 Hz frequency range was analyzed reflecting the dominant frequencies of both SB and NGB events (Supplementary Fig. 9A). This analysis revealed that for most events, the CSC was significantly greater than that for shuffled data across the full range of frequencies, which was most clearly seen for cortex-striatum interactions and during NGB events. These observations were largely consistent with those obtained using imaginary coherence cross-spectral analyses (Supplementary Fig. 9B), which suppresses zero-lag coherence, which also suggested that NGB-NGB co-occurring events are truly coherent across brain regions across most of the frequency range. However, it is not possible to exclude a certain contribution of volume conductance in the measures of coherence amongst SB-SB co-occurring events. Lastly, the analysis of the CSC across development revealed region-specific changes (Fig. 5F). For events in cortex-striatum the CSC appeared mostly stable over time and even slightly increased (SB 0.59 ± 0.11 to 0.70 ± 0.07; NGB: 0.60 ± 0.12 to 0.65 ± 0.08; *F*(3, 1610) = 15.8, *P* = 3.67e-10). In contrast, for events in thalamus-striatum, the coherence declined for both SB and NGB (SB 0.54 ± 0.08 to 0.49 ± 0.10; NGB: 0.58 ± 0.10 to 0.23 ± 0.09; *F*(3, 520) = 27.1, *P* = 2.80e-16), with the largest decrease seen for NGB events (*F*(3, 520) = 8.78, *P* = 1.09e-05). A similar trajectory could be observed for events in cortex-thalamus with an overall decline in CSC during development (SB 0.53 ± 0.07 to 0.46 ± 0.10; NGB: 0.52 ± 0.12 to 0.24 ± 0.10; *F*(3, 538) = 23.5, *P* = 2.71e-14) which was markedly greater for NGB events (*F*(3, 538) = 4.58, *P* = 0.00354).

Overall, these data suggest that although co-occurring bursts in different brain regions are often the same the different brain regions preferentially synchronize during different types of burst events. Furthermore, they suggest that cortex and striatum maintain high levels of synchrony throughout development with evidence for more local generation of burst activity in thalamus, although this possibly might be synchronized to other brain regions not recorded here.

### Cortex mainly drives activity in striatum and thalamus

**Fig. 6:**
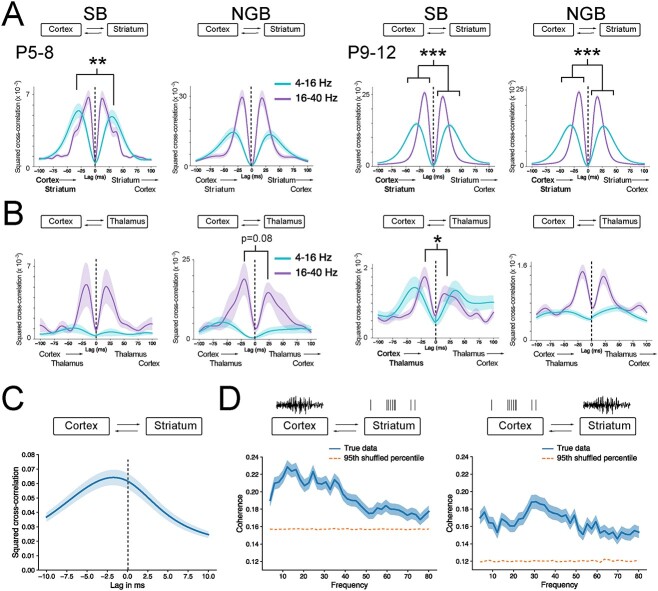
Cortical activity drives activity in downstream basal ganglia. (A) Results from cross-correlation analysis for SB and NGB events that occur within P5–8 and P9–12 in cortex and striatum. Note the overall larger cross-correlation values for interactions between cortex and striatum and the significant lead of cortex driving striatal SB events at P5–8 at lower frequencies (4–16 Hz). At later developmental stages, cortex significantly leads striatum in both frequency bands. (B) Results from cross-correlation analysis for SB and NGB events that occur within P5–8 and P9–12 in cortex and thalamus. Cross-correlation values for interactions between cortex and thalamus were largest for NGB events at P5–8 with a trend toward cortex driving events in thalamus specifically at higher frequencies (16–40 Hz). Overall correlations dropped at later developmental stages but suggest that cortex is still driving activity in thalamus also at P9–12, specifically at higher frequencies (16–40 Hz) and during SB events. (C) Spike train cross-correlation (convolved with a Gaussian kernel) between cortex and striatum suggest a strong drive of cortex on striatal activity (peak of mean = −1.75 ms, one-sample *t*-test, *P* = 4.26e-06). (D) Spike-field coherence for cortico-striatal interactions. Note the significant interaction of the cortex LFP with spiking in the striatum (left, *P* < 0.001) and the significant interaction of the cortical spiking with striatal LFP (right, *P* < 0.001). Dashed lines indicate the 95th percentile range based on null distribution (shuffled data). Individual bursts as detected in all animals are pooled according to type and brain region in (A), (B), (C), and (D).

To determine whether one brain region might be driving the other during these synchronous burst events, a cross-correlation analysis was performed to calculate the possible lags between co-occurring burst events (see Methods and Fig. 6A, B and Supplementary Fig. 10). Frequency bands of interest were taken as 4–16 and 16–40 Hz ranges, around which spectral power was greatest for respectively SB and NGB bursts. To maximize statistical power due to lower numbers of co-occurring events, the data were split into two groups roughly corresponding to the first (P5–8) and second postnatal week (P9–12).

Overall, the interaction between cortex and striatum produced the largest cross-correlation values irrespective of age or type of burst, indicating that activity between cortex and striatum is highly correlated (P5–8 SB max ρ^2^ = 0.00675, P5–8 NGB max ρ^2^ = 0.0297, P9–12 SB max ρ^2^ = 0.0256, P9–12 NGB max ρ^2^ = 0.0243, Fig. 6A), which is consistent with the previous coherence analysis. Interactions between cortex-thalamus also exhibited overall large cross-correlations, especially at P5–8 in the 16–40 Hz range (P5–8 SB max ρ^2^ = 0.00525, P5–8 NGB max ρ^2^ = 0.0189, P9–12 SB max ρ^2^ = 0.00175, P9–12 NGB max ρ^2^ = 0.00148, Fig. 6B), but here the cross-correlation values tended to decrease by P9–12. The interaction between thalamus and striatum produced only small cross-correlation values independent of age or type of burst (Supplementary Fig. 10). Between brain region pairs, there was a clear peak in the 16–40 Hz band centred around +19/−17 ms, consistent with putative monosynaptic interactions amongst all regions (Hartung et al. 2016a, 2016b). In contrast, the 4–16 Hz band was characterized by a broader cross-correlation curve centred around +41/−42 ms. Using the peaks to explore for potential lags between co-occurring burst events can provide evidence for one brain region leading the other and revealed a significant cortical lead for interactions in cortex-striatum and cortex-thalamus, implying a cortical origin to bursting events recorded subcortically. Firstly, for cortex-striatum events at P5–8, there was a significantly larger peak in the 4–16 Hz range for SB events at −29 ms lag (Wilcoxon Rank Sum test, *P* = 0.00293), indicating a putative cortical lead for SB events during early stages of development (Fig. 6A), which was not seen for NGB events at either frequency range. At P9–12, we find a more universal and significant negative lag across both frequency bands for both SB and NGB events in cortex-striatum (SB: 4–16 Hz, −16 ms; SB: 16–40 Hz, −31 ms; NGB: 4–16 Hz, −16 ms; NGB: 16–40 Hz, −31 ms; Wilcoxon Rank Sum test, all *P* ≤ 0.000128), indicating a more general cortical lead of activity in the striatum at later developmental stages (Fig. 6A). These observations were also consistent with those obtained using spike-field coherence analysis (Fig. 6C), spike-density cross-correlation analysis (Fig. 6D), and unit activity analysis (Supplementary Fig. 13), which are less sensitive to potential volume conduction issues and also suggest a strong cortical drive of striatal activity. To further corroborate these findings in a subset of animals a small local cortical injection of lidocaine was made, which revealed that reducing overall activity in cortex led to a significant reduction in locally generated spiking activity in striatum (Supplementary Fig. 14). Secondly, for cortex-thalamus events, there was a trend toward a cortical lead at P5–8 for NGB events in the 16–40 Hz band with a − 20 ms lag, though this comparison did not reach significance (Wilcoxon Rank Sum test, *P* = 0.0803), but at later developmental stages (P9–12), some evidence was found for a cortical lead for SB events at −19 ms lag (Wilcoxon rank sum test, *P* = 0.0406, Fig. 6B). The spike-field coherence analysis also suggested a significant interaction between cortical spiking and thalamic LFP (Supplementary Fig. 11A), but the spike-density cross-correlation analysis was less clear (Supplementary Fig. 12A). Finally, there was no evidence for a significant lead in the peaks for thalamus-striatum events (Wilcoxon rank sum test, all *P* > 0.05), suggesting that neither region was responsible in clearly driving burst events (Supplementary Fig. 10), which was also corroborated by further spike-field coherence (Supplementary Fig. 11B) and spike-density cross-correlation analysis (Supplementary Fig. 12B).

Together, these results suggest that from all possible interactions during bursts of activity between brain regions the synchronization of activity between cortex and striatum and cortex and thalamus is greatest. Furthermore, they suggest that activity in cortex can drive activity in these two subcortical nuclei, which is already apparent during the first postnatal week and seen within lower frequency bands and become stronger and more pronounced during the second postnatal week at both frequency bands.

## Discussion

Here we describe for the first time the bursts of neural activity that occur within key nodes of the developing mouse basal ganglia, including the cortical and thalamic input structures and the dorsal striatum. By means of an unbiased detection and classification approach, we demonstrate that network bursts can be broadly classified into two different types and include SB and NGB, which are observed in all three brain regions. The analysis of these bursts across postnatal days (P)5 to P12 suggests that these patterns of activity exhibit many distinct developmental changes that appear in parallel in each brain region, including developmental shifts from predominant SB to NGB events, amongst others. Many of the bursts tended to occur synchronously in the different brain regions and were often, but not always, of the same type. Both coherence and cross-correlation analyses suggested different levels of interactions during SB to NGB events with the strongest interactions between cortex and striatum as compared with other brain regions. Significant interactions could already be observed during the first postnatal week, and these became stronger during the second postnatal week and were consistent with activity in cortex driving activity in striatum and to some extent thalamus, initially apparent within lower frequency bands (4–16 Hz) and later in development apparent also within higher frequency bands (16–40 Hz).

Network bursts were recorded in urethane anesthetized C57 mice during the first postnatal weeks using multi-channel silicon probes. This anesthetic regime is thought to help preserve naturalistic activity dynamics (Sitdikova et al. 2014; Shumkova et al. 2021) akin to a sleep state and therefore reflective of the default state of mouse pups during this developmental period (Bolles and Woods 1964; Clement et al. 2008). In general, neonatal bursts are typically classified based on their structural and spectral properties, and in developing cortex, these include “spindle bursts” as recorded in rodents (Khazipov et al. 2004; Hanganu et al. 2006; Yang et al. 2016), and their human equivalent “delta-brushes” as recorded from preterm human neonates (Vanhatalo and Kaila 2006; Milh et al. 2007; Vecchierini et al. 2007). These are thought to be one of the earliest activity patterns generated in the young postnatal brain—but see for early prenatal thalamic activity patterns (Moreno-Juan et al. 2017; Anton-Bolanos et al. 2019), and consist of short bursts of activity containing 4–16 Hz oscillations that can be triggered by distal muscle twitches and are thought to contribute to the establishment of circuits required for sensorimotor coordination (Khazipov et al. 2004; Milh et al. 2007; Dooley and Blumberg 2018). In addition, a second major pattern of activity, so-called “gamma bursts,” also co-occurs in cortex during the first postnatal weeks (Yang et al. 2009; Minlebaev et al. 2011; Khazipov et al. 2013; Yang et al. 2013) and consist of short bursts of activity in the gamma frequency range (20–40 Hz). Both patterns of activity can result from peripheral activity such as whisker activation, muscle twitches, or retinal waves and are often topographically organized according to the site of sensory stimulation (Yang et al. 2013; Gerasimova et al. 2014), but can also arise spontaneously from the intrinsic dynamics within the developing circuits (Hanganu et al. 2006; Yang et al. 2013; Luhmann et al. 2016; Martini et al. 2021). These early bursts of activity are largely transient and disappear at later stages of development and are replaced by persistent oscillatory activity, such as gamma oscillations (Khazipov et al. 2013). Often, these early bursting patterns do not occur in isolation but rather form complex, nested motifs. For example, transient gamma oscillations often precede SB (Yang et al. 2013) or can be nested within SB (Brockmann et al. 2011), and both SB and gamma oscillations might be nested within larger delta waves (Khazipov et al. 2013).

Overall, the literature on neonatal bursting activity is highly diverse, even when restricted to studies in rodent models and similar brain regions (Yang et al. 2009; Seelke and Blumberg 2010; Brockmann et al. 2011; An et al. 2014). This diversity of findings likely results from a combination of factors, including exact recording protocols, differing levels of anesthetics and/or recording ages, both species-specific and exact recording location-specific factors, and the lack of standardization for classifying and naming of bursting events. Therefore, we opted for an unbiased detection and classification approach for these recordings. Using this approach, we suggest that two distinct classes of bursting activity can be reliably identified across cortex, striatum, and thalamus that we refer to as SB and NGB as these exhibited properties very similar to those described previously in cortical regions, including their spectral/oscillatory properties with a peak for SB at 4–16 Hz and for NGB an additional faster frequency peak at 20–30 Hz (Contreras et al. 1997; Khazipov et al. 2004; Hanganu et al. 2006; Yang et al. 2009, 2013; An et al. 2014; Cichon et al. 2014; Gerasimova et al. 2014; Hartung et al. 2016a; Bitzenhofer et al. 2017; Murata and Colonnese 2018; Chini et al. 2020). However, subtle differences were observed in the properties of events in our recordings made at the border of the motor and somatosensory cortex and those described in other cortical structures. For example, the duration of NGB events in our recordings were predominantly longer than SB events, similar to those seen in more frontal cortical regions (Brockmann et al. 2011), but differing from those recorded in sensory cortical areas where bursts of gamma oscillations were significantly shorter than SB events (Yang et al. 2009). Although it is unclear whether similar bursting events share a common underlying cellular mechanism of generation in different brain regions (Hanganu-Opatz et al. 2021), it is possible that differences in cellular and circuit architecture across visual, somatosensory, and prefrontal cortex (Allene et al. 2008; Katzel et al. 2011; Tasic et al. 2018) might contribute to these observed differences. The bursts recorded and analyzed in this dataset were largely similar amongst brain regions while also exhibiting region-specific differences. For example, during NGB bursts, the overall power of the beta-gamma oscillations was greatest in cortex, the spike rate was greatest in thalamus, and the peak frequency of theta-alpha oscillations differed between brain regions ranging from 7 Hz for cortex and striatum to 14 Hz in thalamus. These differences likely reflect a combination of region-specific network dynamics, overall levels of neural activity, and precise positioning of current sinks and sources (Tanaka and Nakamura 2019). Although anatomical recording positions were kept as constant as possible between different mice and across ages, the possibility cannot be exclude that recordings could be impacted by developmental shifts in cortical or thalamic axonal innervation and dissimilarities in the composition of the striatal microcircuits, for example, those between lateral and medial striatum (Gerfen et al. 1985; Matamales et al. 2016; Munoz-Manchado et al. 2018; Alegre et al. 2021).

The analysis of the changes in these bursts across development suggests a few interesting developmental trajectories for both SB and NGB events that were evident in all brain regions studied. For example, the NGB events tended to increase in incidence, whereas SB events reduced in incidence across development. These developmental trajectories are consistent with the wider literature where spindle activity tends to decline with age (Nakazawa et al. 2020), whereas gamma bursts (Seelke and Blumberg 2010) and gamma-band activity more generally increases over time (Minlebaev et al. 2011; Bitzenhofer et al. 2020). These changes in event incidence likely reflect progressive maturation of neuronal circuits able to sustain faster frequency events and for longer periods of time. Within the cortex, gamma-band activity is thought to depend on interactions between interneurons—in particular perisomatic-targeting parvalbumin-positive cells—and pyramidal cells, where the reciprocal excitatory and inhibitory interactions give rise to gamma frequency oscillations (Tiesinga and Sejnowski 2009). Thus, the developmental increase in gamma-band activity seen during NGB events likely reflects the integration of perisomatic interneuron-mediated inhibition within glutamatergic circuits (Khazipov et al. 2013). Recordings of larger numbers of units and using waveform analysis to classify neuronal populations could help to investigate when interneurons in the basal ganglia and afferent structures come “online” and how they contribute to oscillatory activity (Reyes-Puerta et al. 2015; Bitzenhofer et al. 2020). In parallel, the progressive maturation of intrinsic electrical properties of principal neurons and the synaptic connections between striatum and its afferent structures will also be able to support higher frequency activity (Krajeski et al. 2019). The absence of mature ionic currents in neurons at these young ages might also explain the observations that in neonatal thalamus bursts were seen containing ~ 5–20 action potentials at a frequency of around 5–30 Hz, as opposed to higher frequencies (>150 Hz) seen during low-threshold bursts in the CL intralaminar thalamic nuclei in adulthood (Lacey et al. 2007). However, many of these changes in cellular and circuit properties that underlie the observed developmental changes in oscillatory behavior within the basal ganglia remain largely unknown.

The main excitatory afferents to the mature striatum arise from neurons located in the cortex and thalamus (Kemp and Powell 1971; Buchwald et al. 1973; Smith et al. 2004). The main thalamic afferents to the striatum originate in the (higher order) intralaminar nuclei (Macchi et al. 1984; Berendse and Groenewegen 1990; Castle et al. 2005; Smith et al. 2009; Ellender et al. 2013). These cortical and thalamic axons and synapses are both present and functional in the striatum in the first postnatal week and onwards (Nakamura et al. 2005; Doig et al. 2010; Ellender et al. 2013; Smith et al. 2014; Sohur et al. 2014; Krajeski et al. 2019), for example, as evidenced from spontaneous and electrically excitatory postsynaptic currents (EPSCs; Peixoto et al. 2016; Krajeski et al. 2019). This suggests that the anatomical substrate is in place for early network activity as recorded here to propagate to striatum, but these synapses exhibit dynamic changes in both strength and number well into the second postnatal week and onwards (Butler et al. 1998; Sharpe and Tepper 1998; Tepper et al. 1998; Dehorter et al. 2011; Peixoto et al. 2016; Krajeski et al. 2019). These include changes in glutamate receptor composition and short-term synaptic plasticity, which will impact the propagation of activity (Ellender et al. 2011; Ellender et al. 2013; Krajeski et al. 2019). For example, a faster rate of maturation of AMPA receptor-mediated transmission was observed for thalamostriatal synapses as compared with corticostriatal synapses in the first postnatal weeks (Krajeski et al. 2019). Indeed, this might explain observations made here of rapid changes in thalamus-striatum interactions from the first to second postnatal week (e.g. mean cross-spectral coherence). However, as both cortical and thalamic synapses are present and functional from the first postnatal week onwards, at least on SPNs (Krajeski et al. 2019), this does not explain why cortex appears to drive activity more strongly in striatum as compared with thalamus. It would be interesting to investigate to what extent this might be the result of differential recruitment of certain classes of striatal interneurons during early stages of development (Assous et al. 2017; Tepper et al. 2018) as these are able to exhibit strong control of striatal oscillatory activity (Sharott et al. 2009). The intralaminar thalamic nuclei also provide inputs to the cortex in adulthood which are reciprocal (Saalmann 2014) but these inputs are relatively minor (Sadikot et al. 1992a; Parent and Parent 2005), which might help explain the relatively lower levels of interactions we observe here between cortex-thalamus. However, much here is not known for rodents or during early development (Smith et al. 2014; Smith et al. 2022), but our results would suggest such interactions are significantly reduced as compared with sensory thalamus (Martini et al. 2021).

The analysis of the interactions amongst brain regions during burst activity in neonatal and young mice revealed several interesting dynamic changes. First, the events that co-occurred amongst brain regions often were of the same type and that such interactions increased over developmental time. This likely reflects maturation of direct synaptic connections amongst the brain regions (Tepper et al. 1998; Nakamura et al. 2005; Dehorter et al. 2011; Peixoto et al. 2016; Krajeski et al. 2019). Secondly, that the coherence between brain regions was greatest between cortex and striatum and that this coherence was retained throughout the first and second postnatal weeks. This is consistent with the observation that in adulthood also the activity within striatum is strongly reflective of activity within cortex (Peters et al. 2021), as propagated by the extensive excitatory cortico-striatal afferents (Hunnicutt et al. 2016). The coherence between cortex and thalamus and thalamus and striatum was initially also high but this decreased significantly across development, suggesting progressive uncoupling and/or increased local generation of these burst events. Thirdly, cross-correlation analysis of co-occurring bursts in different brain regions would suggest that cortex significantly leads activity in striatum both during the first and second postnatal weeks, initially seen during lower frequencies and later also during faster frequencies of activity. In addition, the cortex seems to lead activity in thalamus to some extent as well, but this was only evident during later developmental stages and restricted during faster frequencies of activity. One caveat in any measurements of coherence and synchrony in small brain structures is that suggested interactions could be impacted by volume conductance (although many recorded bursts (30–40%) appeared in isolation within one brain region only). To address this imaginary coherence, spike-field coherence and spike-density cross-correlation analysis was used, which would suggest that functional interactions do occur between brain regions, as opposed to resulting from volume conduction only (Boraud et al. 2005), with the strongest evidence for interactions seen during NGB-NGB events in cortex and striatum during the first postnatal weeks. The spike-field coherence and spike-density cross-correlation analyses are based on recorded action potentials and this activity is inherently very local in origin.

The weakest interactions were seen for activity between striatum and thalamus which suggest that during this developmental period these brain regions do not strongly interact directly and that burst of synchronous activity might be led by a tertiary structure—for example, a common cortical area or areas. Indeed, as SB have been shown to propagate across diverse cortical regions (An et al. 2014) and even across hemispheres (Yang et al. 2009), the possibility of an extended cortical driver via long-range connections is plausible. Indeed, this is also supported by experiments using small injections of lidocaine within cortex, which had the most pronounced and significant effect on spiking in striatum but appeared to reduce activity in thalamus also. More precise and selective inactivation of certain brain regions (e.g. via optogenetic stimulation; Bitzenhofer et al. 2017) would provide more conclusive evidence for which regions are driving others. However, as LFP signals arise from the summation of many aligned voltage sources and are expected to emerge more clearly from organized and laminar structures (e.g. cortex), as opposed to more homogeneous structures such as the basal ganglia (Buzsaki et al. 2012), it could be that recordings of striatal and thalamic LFP and spikes is not best placed to reveal interactions.

In conclusion, we find that the developing basal ganglia exhibits distinct oscillatory activity patterns during the first postnatal weeks, which can be classified as either SB or NGB. These appear to be predominantly initiated in cortical regions and drive activity in downstream nuclei. Although it has been shown that neural activity in general is required for maturation and survival of striatal neurons (De Marco Garcia et al. 2011; Kozorovitskiy et al. 2012; Peixoto et al. 2016; Peixoto et al. 2019; Sreenivasan et al. 2022), it is tempting to speculate that these different types of bursts have different functional roles during early postnatal development. Indeed, they might convey distinct developmentally relevant information to the basal ganglia as well as contribute toward the establishment of its fine-scale synaptic connectivity by engaging distinct synaptic plasticity rules (Reynolds et al. 2001; Kozorovitskiy et al. 2015; Valtcheva et al. 2017; Han et al. 2020; Mendes et al. 2020). Taken together, these results demonstrate the dynamic nature of burst generation within the developing basal ganglia and aims to facilitate future explorations of the physiological roles for these bursts in its development.

## Supplementary Material

REVISION_In_vivo_recording_of_network_activity_SUPPLEMENTAL_Cere_bhad307
