# Supplementary material for: Generation and propagation of bursts of activity in the developing basal ganglia
Source: Cereb Cortex. 2023 Aug 23;33(20):10595–613. doi: 10.1093/cercor/bhad307 (PMC10560579; doi:10.1093/cercor/bhad307)
Supplement: REVISION_In_vivo_recording_of_network_activity_SUPPLEMENTAL_Cere_bhad307 [file revision_in_vivo_recording_of_network_activity_supplemental_cere_bhad307.docx]

**Supplemental Material**

**Supplemental Figures + Tables**

**Klavinskis-Whiting *et al.* 2023 – Revision/Typeset**

**Number of Supplemental Pages:** 29

**Number of Supplemental Figures:** 15

**Number of Supplemental Tables:** 12

**Supplemental Figures**

**
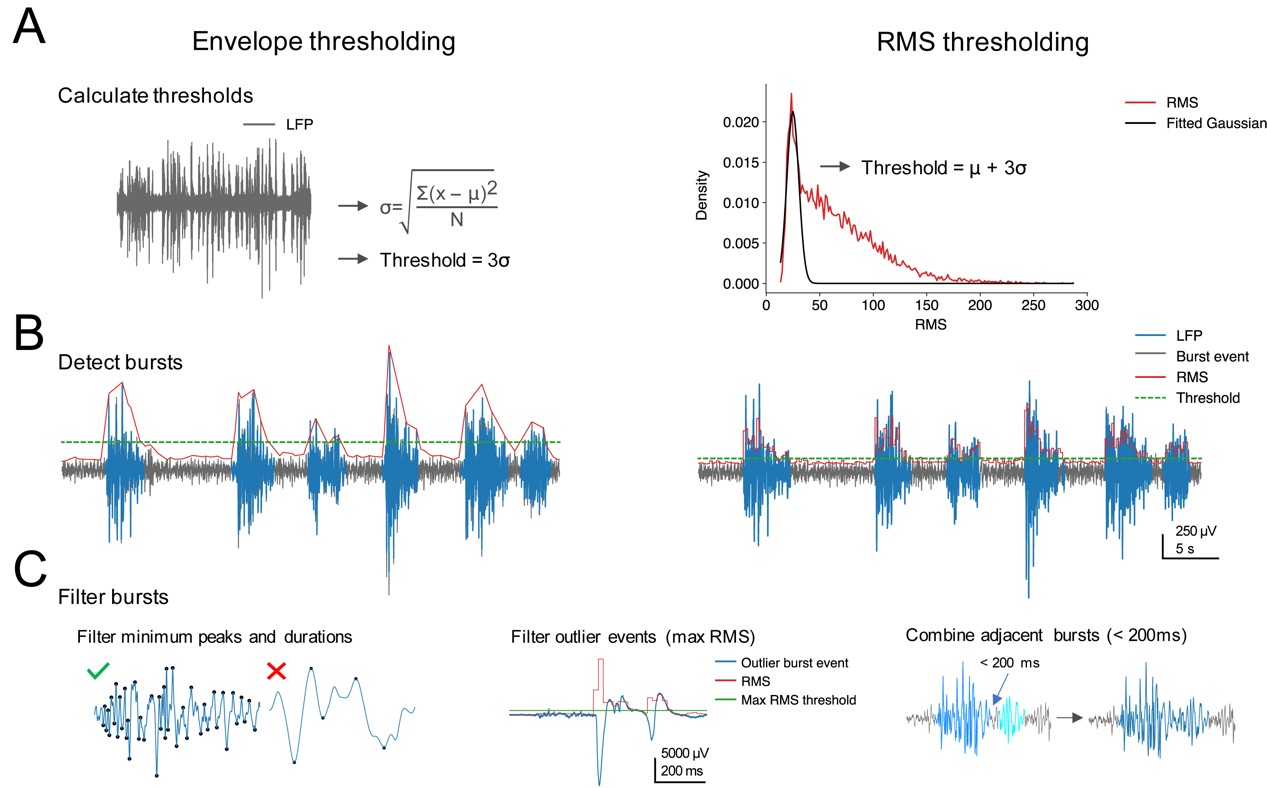
**

**Supplemental Figure 1: Burst detection algorithms used for analysis of recordings.** (**A**) Two different algorithms were initially trialled. The bursting threshold was first computed either based on the standard deviation of the LFP signal (Envelope thresholding, left) or on the mean and standard deviation parameters of the fitted Gaussian (RMS thresholding, right). See also **Methods**. The RMS thresholding method was chosen for subsequent analyses as it is better validated in existing literature while being more parsimonious insofar as it does not require any per-recording parameters to be decided by the experimenter. (**B**) Burst events (in blue) were defined as those periods exceeding the threshold. (**C**) Putative burst events were subsequently checked to ensure they adhered to a minimum duration and number of peaks, while not exceeding the outlier RMS threshold. Finally, adjacent detected bursts found within 200 ms were deemed to be part of one burst event and combined.

**
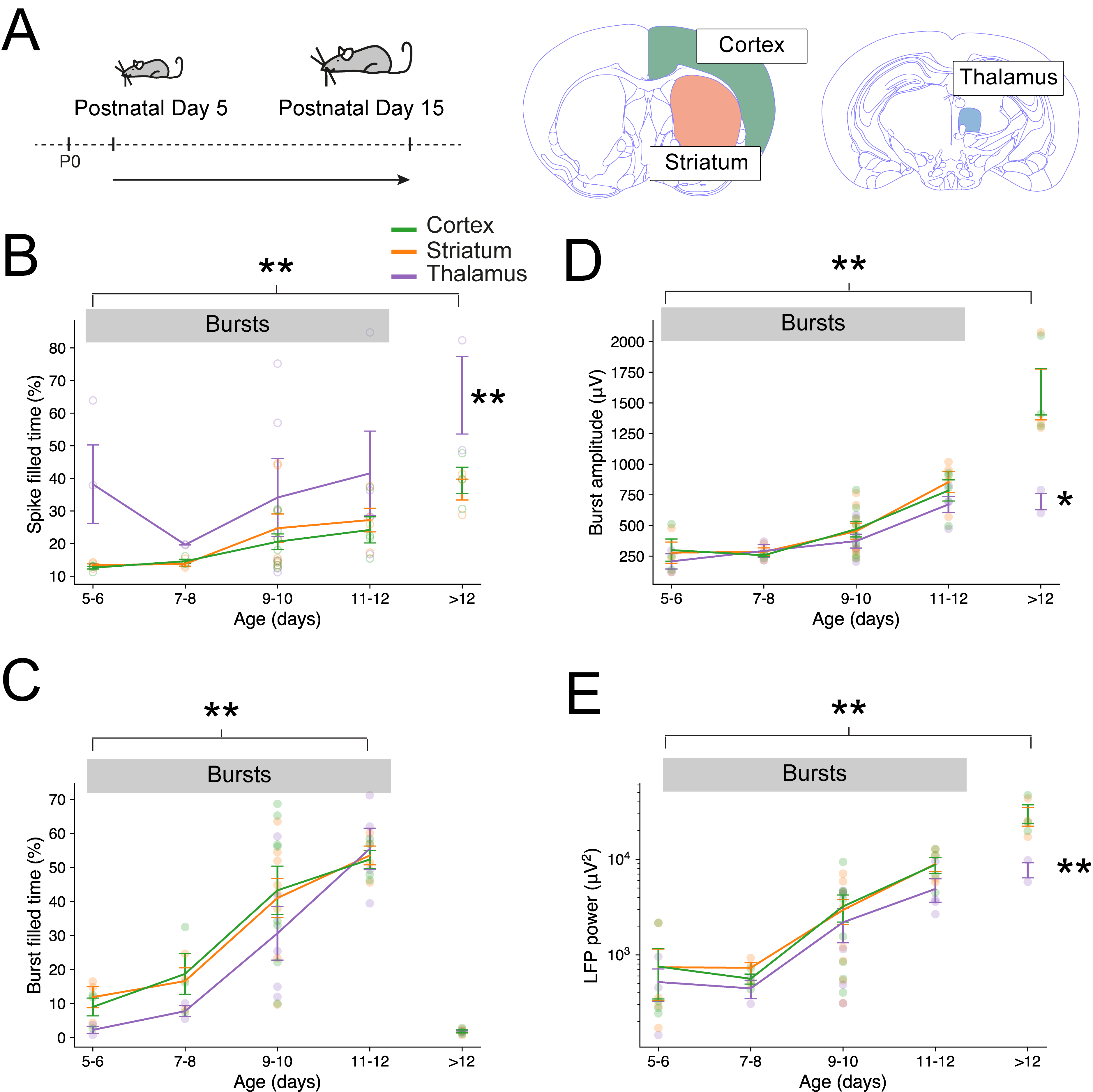
**

**Supplemental Figure 2: General increase in neural activity in all three brain regions across postnatal development.** (**A**) Recordings were made from the cortex, dorsal striatum and intralaminar thalamus in mice between postnatal days (P)5-15. (**B**) Spike-filled time; fraction within 200 ms recording windows containing at least one spike event, increased significantly across postnatal development (F(4,45) = 4.66, p = 0.00312), while there was also a significant effect of group whereby spike-filled time was greater for the thalamus (F(2, 45) = 6.78, p = 0.00270). (**C**) Burst-filled time; proportion of time within each recording occupied by bursting events also increased significantly across postnatal development (F(4,42) = 26.1, p = 6.72e-11), before the main activity in all three brain regions transitioned to a continuous oscillatory pattern. (**D**) Mean burst amplitude, defined as the difference between the minimum and maximum peaks in the 4 -100 Hz bandpass filtered LFP signals for each bursting event, increased significantly across postnatal development (F(4, 42) = 42.1, p = 3.61e-14). There was also a main effect of brain area (F(2, 42) = 4.88, p = 0.0125), where amplitude was lower across the thalamus, and a significant interaction effect of age and brain area (F(8, 42) = 2.50, p = 0.0258), likely driven by the disparity in amplitude at later developmental ages (> P12). (**E**) Local field potential (LFP) power in 4-100 Hz increased significantly across postnatal development (F(4,45) = 37.0, p = 1.06e-13). There was also a main effect of brain area (F(2, 45) = 4.21, p = 0.0212), where LFP power was lower for thalamus than for cortex and striatum, and a significant interaction effect of age and brain area (F(8, 45) = 3.07, p = 0.00754), where the increase in LFP power over time for thalamus was less pronounced as compared to cortex and striatum. All bursts are pooled across animals and split according to age.

**
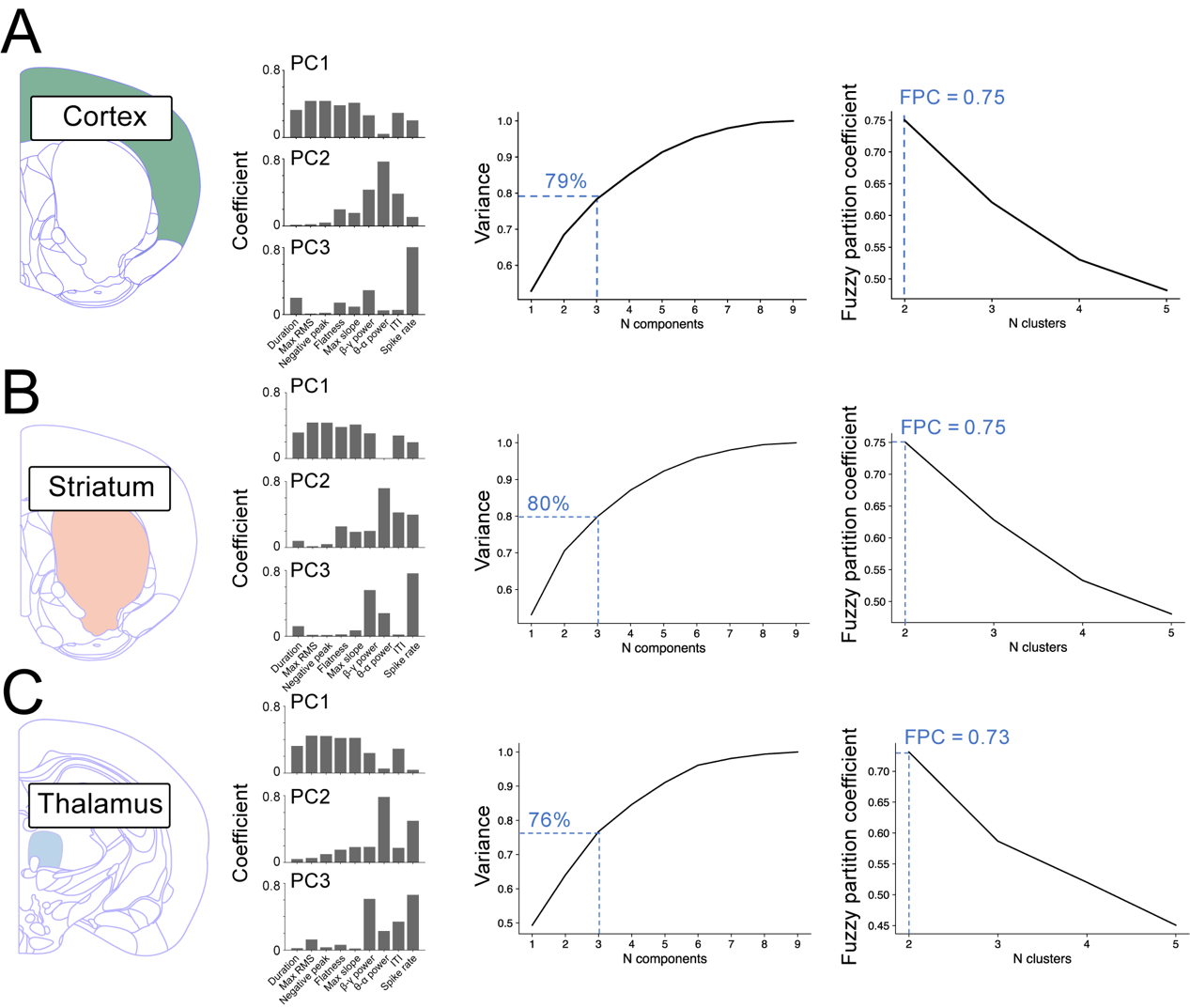
**

**Supplemental Figure 3: Principal component analysis (PCA) of detected events.** Coefficient contribution of each feature to the first three principal components of detected (**A**) cortical, (**B**) striatal and (**C**) thalamic bursts and explained variance (middle) and fuzzy partition coefficients (right) of detected bursts. Considering the contribution of different features to the first three principal components, PC1 appeared to represent a relatively uniform contribution across all features, albeit with minimal contribution of theta-alpha power. PC2 and PC3 were more dominated by specific features — namely theta-alpha power for PC2 and spike rate for PC3. Overall results were comparable across brain regions, though the explained variance for the first three components was slightly lower for the thalamic (76%) as compared with cortical (79%) and striatal (80%) burst events.

**
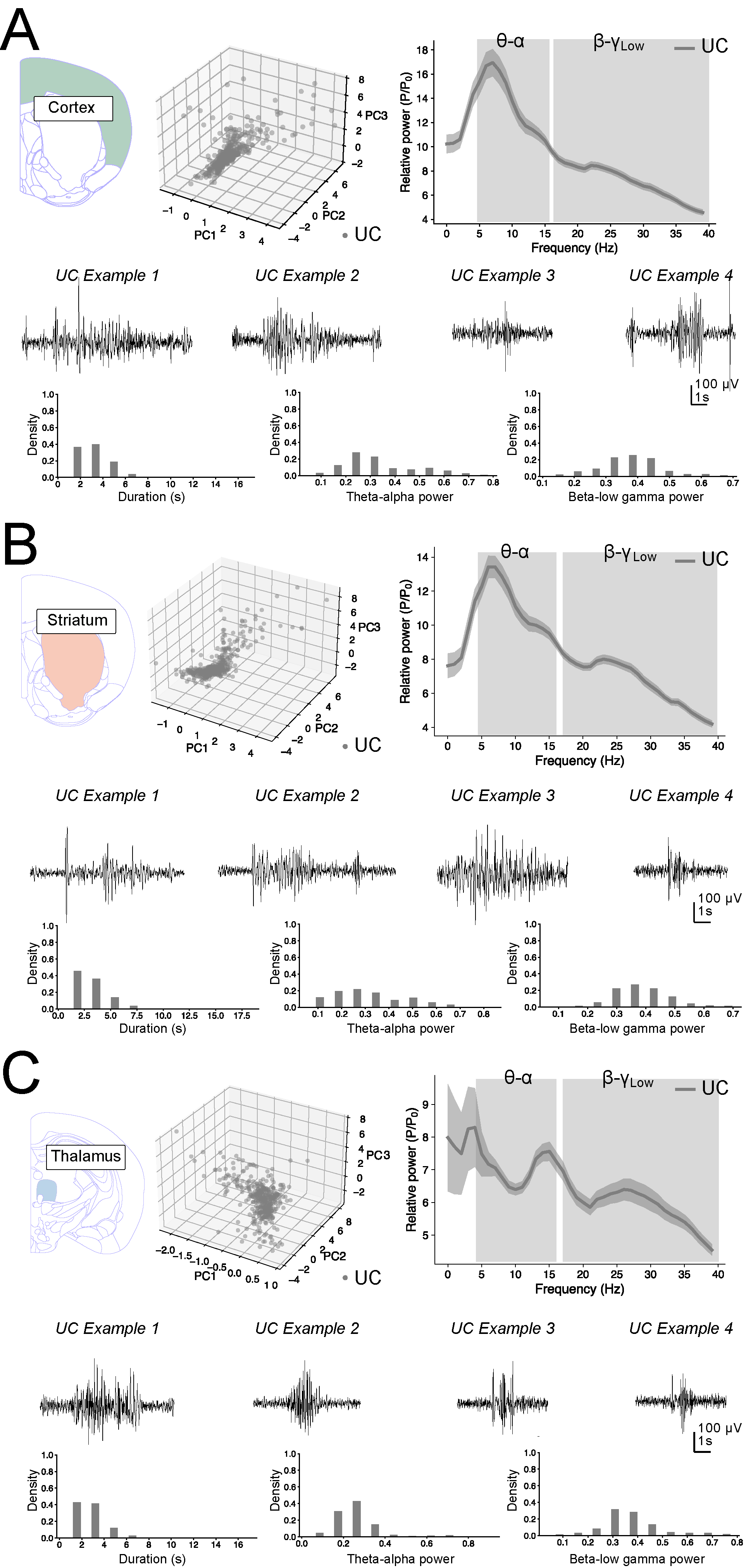
**

**Supplemental Figure 4: Characteristics of unclassified (UC) burst events.** (**A**) Scatter plot of the first three principal components of cortical unclassified (UC) bursts (top, left). PSD of the mean normalized power across UC events (top, right). Note the prominent peak in the theta-alpha frequency range (4-16 Hz). Four example UC events in striatum (middle). Histogram of the distributions of several key features across UC events including their duration, theta-alpha (θ-α) power, and beta-low gamma (16-30 Hz, β-γ_Low_) power (bottom). (**B**) Similar for UC burst events in striatum and (**C**) UC burst events in thalamus. Individual bursts are pooled across all animals and ages in A, B and C.

**
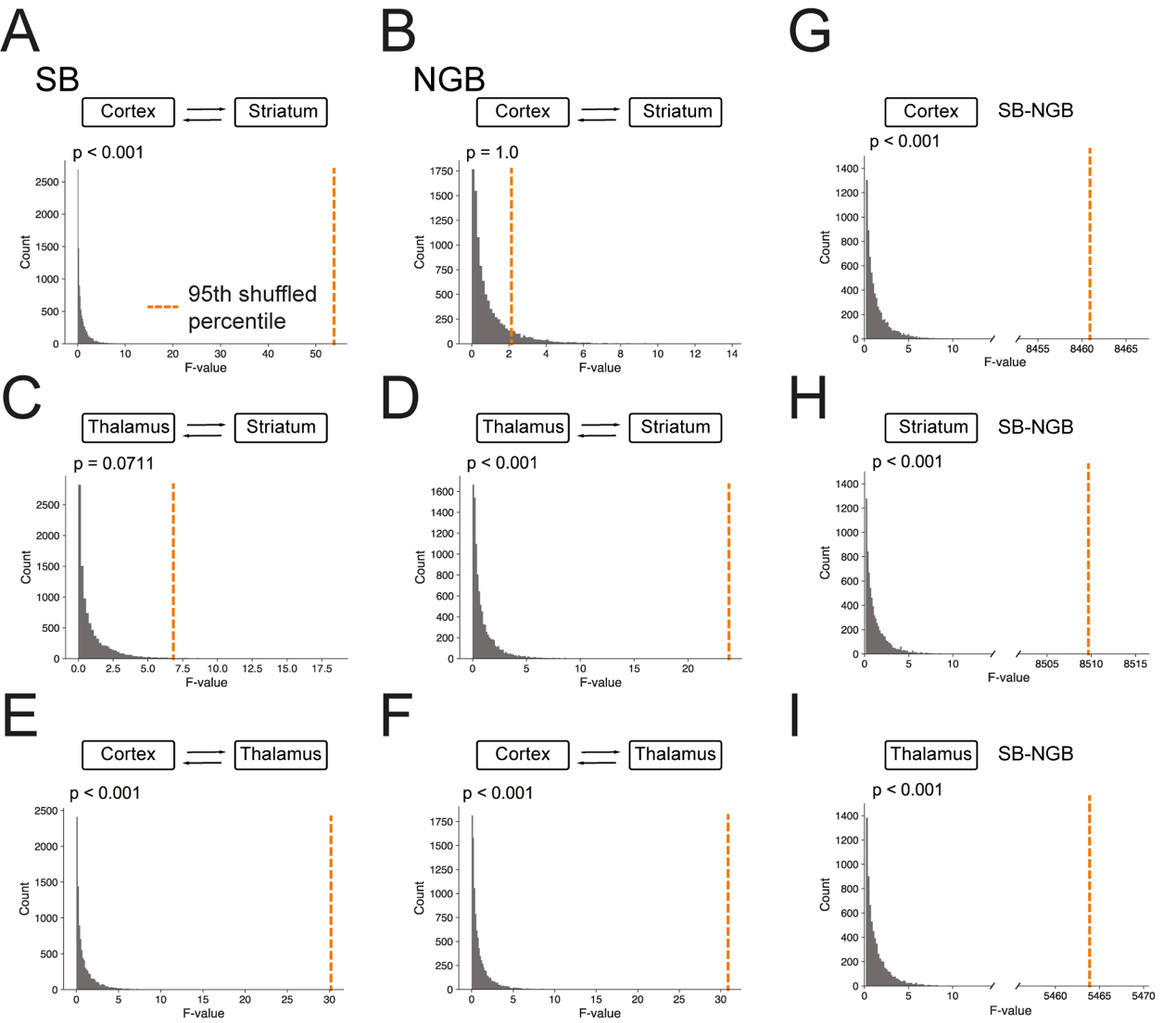
**

**Supplemental Figure 5: F-ratio comparison testing for NGB and SB events.** The significance was determined by shuffling the feature vectors between the two groups 10,000x and calculating the resulting F-statistic at each iteration to produce a null distribution. The significance threshold was defined as the 95^th^ percentile of this null distribution (orange dashed line), with p-values given here corrected for multiple comparisons. There was a significant effect of brain area across all comparisons (**A, D, E, F**) except for NGB events between the cortex and striatum (F(1, 4477) = 2.13, adjusted p = 1.0, **B**) as well as SB events between the striatum and thalamus (F(1, 3624) = 6.82, adjusted p = 0.0711, **C**), which did not reach significance after correction for multiple comparisons. F-ratio test for burst events across (**G**) cortex, (**H**) striatum and (**I**) thalamus. The F-ratios are large for cortex, striatum as well as thalamus, indicating a robust effect of burst type. Individual bursts are pooled across all animals and ages and split according to brain region.

**
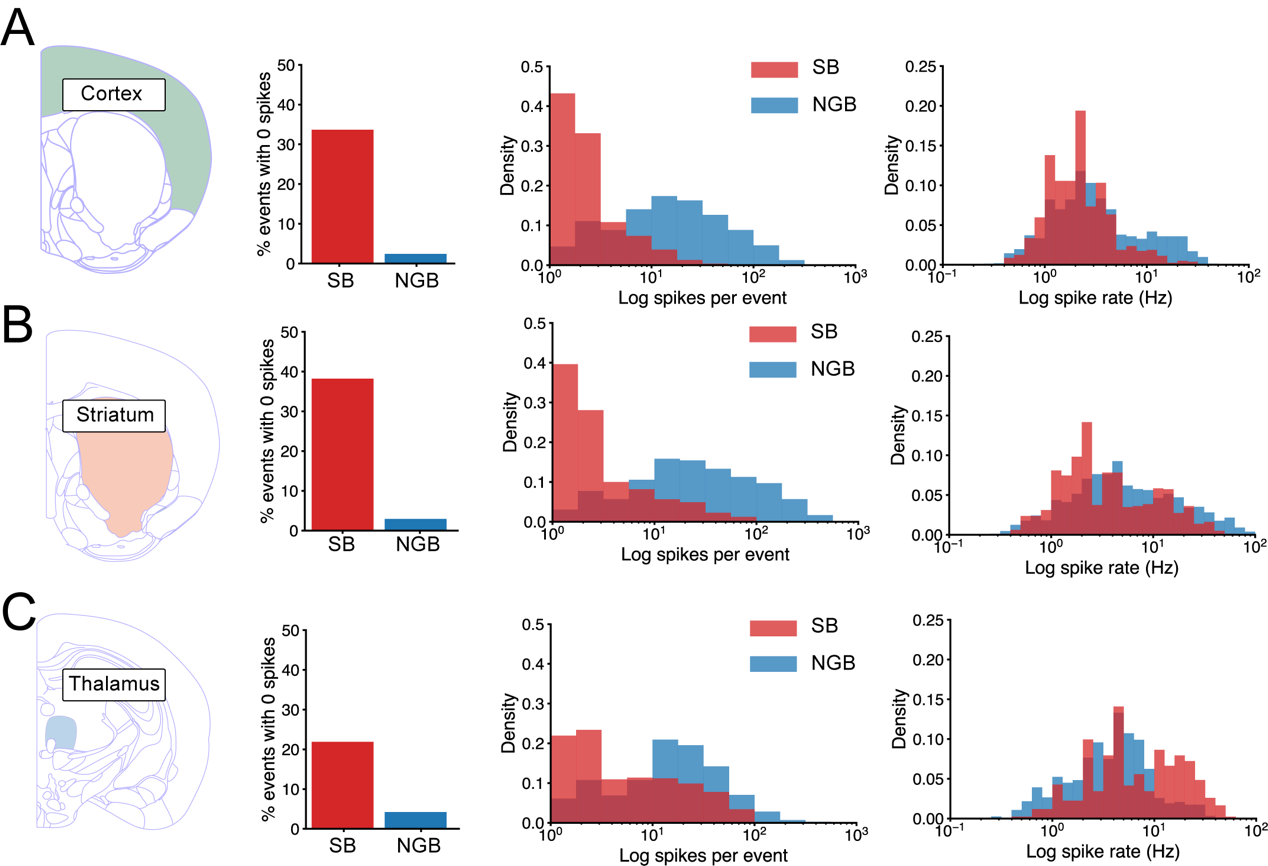
**

**Supplemental Figure 6: Characteristics of MUA activity within bursts.** (**A**) The proportion of burst events in cortex that did not contain significant spiking was greatest during SB events (left). For those events that did contain spikes the distribution the number of spikes detected (middle). Note the higher number of spikes seen during NGB events. The distribution of spike frequencies in burst events (right). Note the similar frequencies of spikes between SB and NGB events. Similar analysis and results for (**B**) striatum and (**C**) thalamus. Not in thalamus that the higher frequency of spikes was observed during SB events. MUA within distinct bursts are pooled across all animals and ages and split according to brain region.

**
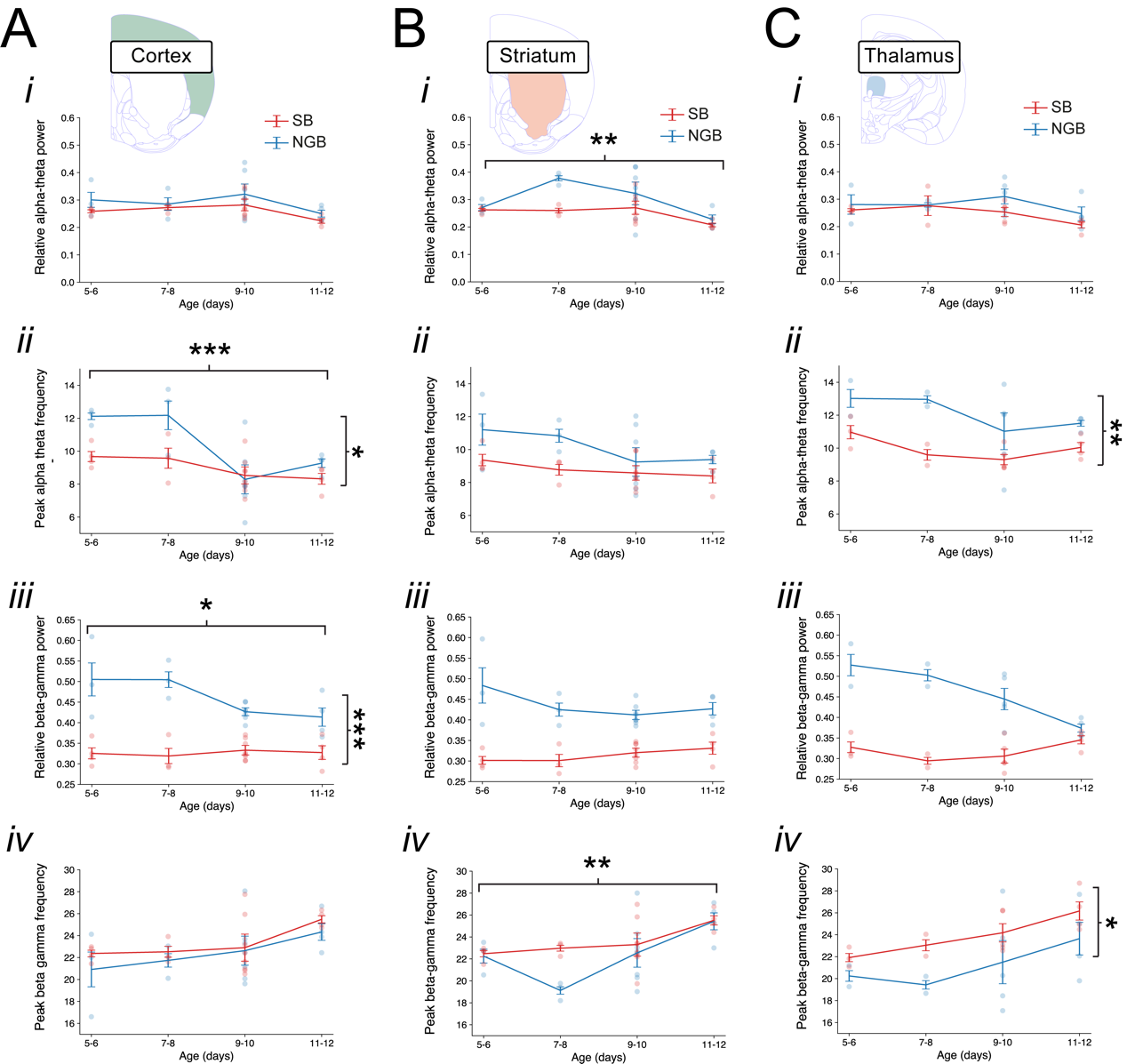
**

**Supplemental Figure 7: Developmental changes in burst properties.** (**A**) Changes in the peak theta-alpha power (*i*) and frequency (*ii*) and peak beta-gamma power (*iii*) and frequency (*iv*) of those occurring in cortex. (**B**) Changes in the peak theta-alpha power (*i*) frequency (*ii*) and peak beta-low gamma power (*iii*) frequency (*iv*) of those occurring in striatum. The theta-alpha frequency component within striatal bursts across development exhibited a decrease in power (F(3*,*29) = 5*.*32*,* p = 0.00480) while the peak frequency did not change (F(3*,*29) = 2*.*21*,* p = 0.108). In contrast, the beta-low gamma frequency component within striatal bursts across development remained constant in power (F(3*,*29) = 2*.*34*,* p = 0.564), but significantly increased in frequency (F(3*,*29) = 6*.*56*,* p = 0.00160) increasing from 22.4 Hz to 25.5 Hz. (**C**) Changes in the peak theta-alpha power (*i*) frequency (*ii*) and peak beta-low gamma power (*iii*) frequency (*iv*) of those occurring in thalamus. Both the theta-alpha frequency and beta-low gamma frequency components within thalamic bursts remained constant in power (theta-alpha: F(3*,*19) = 1*.*86*,* p = 0.171 and beta-low gamma: F(3*,*19) = 2*.*54*,* p = 0.0752) and frequency (theta-alpha: F(3*,*19) = 2*.*20*,* p = 0.121 and beta-low gamma: F(3*,*19) = 2*.*69*,* p = 0.0752) across development. Note that the peak theta-alpha frequency was greater for NGB over SB events (F(1, 19) = 14.2, p = 0.00131) and the peak beta-low gamma frequency slightly faster for SB than NGB events (F(1, 19 = 5.93, p = 0.0249), though the overall power in these SB events was lower (F(1, 19) = 64.8, p = 1.54e-07). Interestingly, NGB beta-low gamma power appeared to decrease while remaining constant for SB events over time (F(3, 19) = 6.52, p = 0.00323). Individual bursts were pooled across all animals, split according to type and plotted according to age.

**
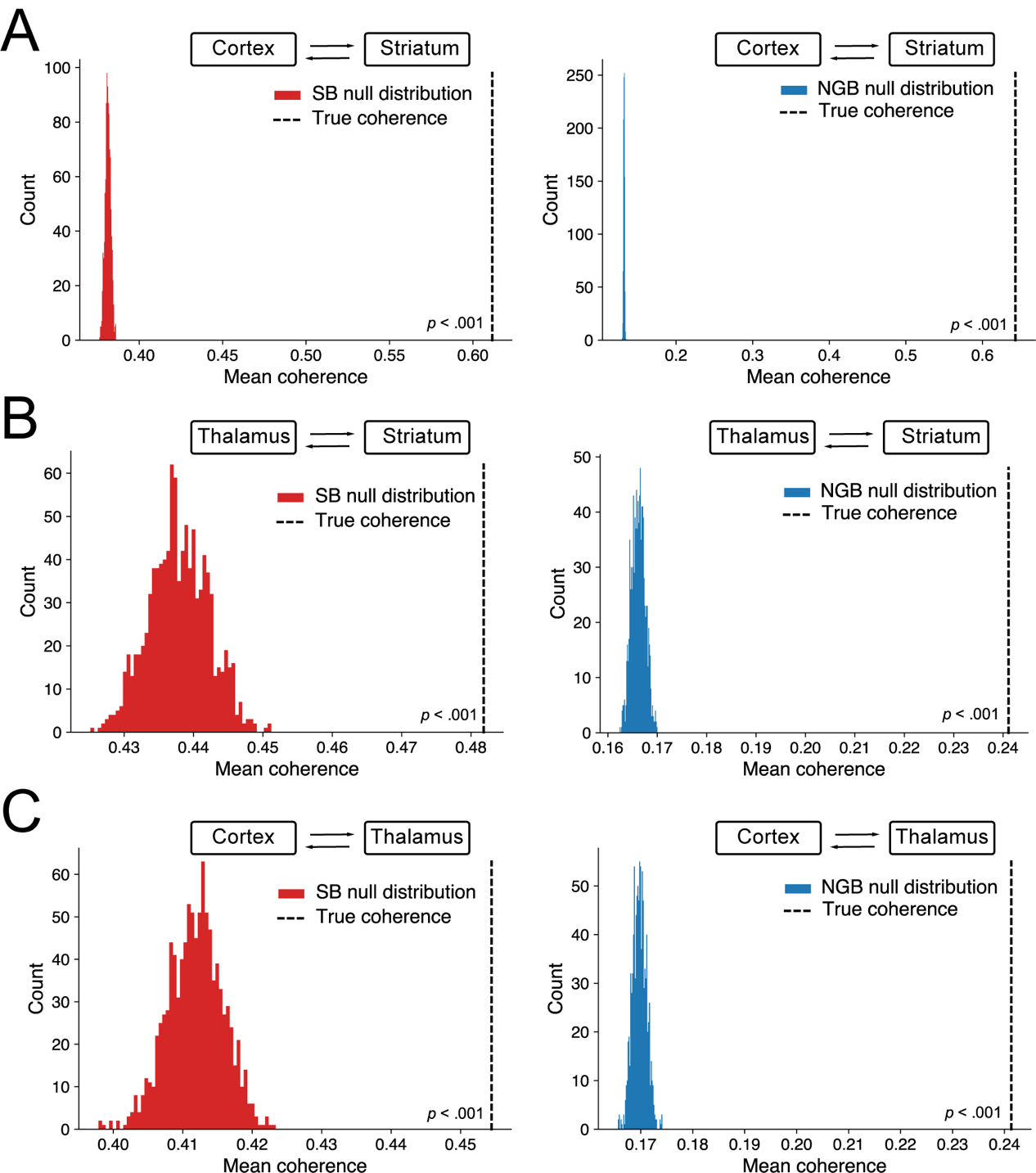
**

**Supplemental Figure 8: Permutation test for mean cross-spectral coherence across SB and NGB events.** (**A**) Pairs of cortical-striatal events. (**B**) Pairs of thalamic-striatal events. (**C**) Pairs of cortical-thalamic events. Pairs of NGB and SB events were randomly shuffled 1000 times, with the mean coherence across all shuffled pairs computed on each iteration to produce a null distribution. The significance threshold was defined as the 95^th^ percentile of this null distribution. Note that for all events the mean cross-spectral coherence was significantly greater for SB and NGB pairs compared to randomly shuffled data. Individual bursts were pooled across all animals and ages and split according to type.

**
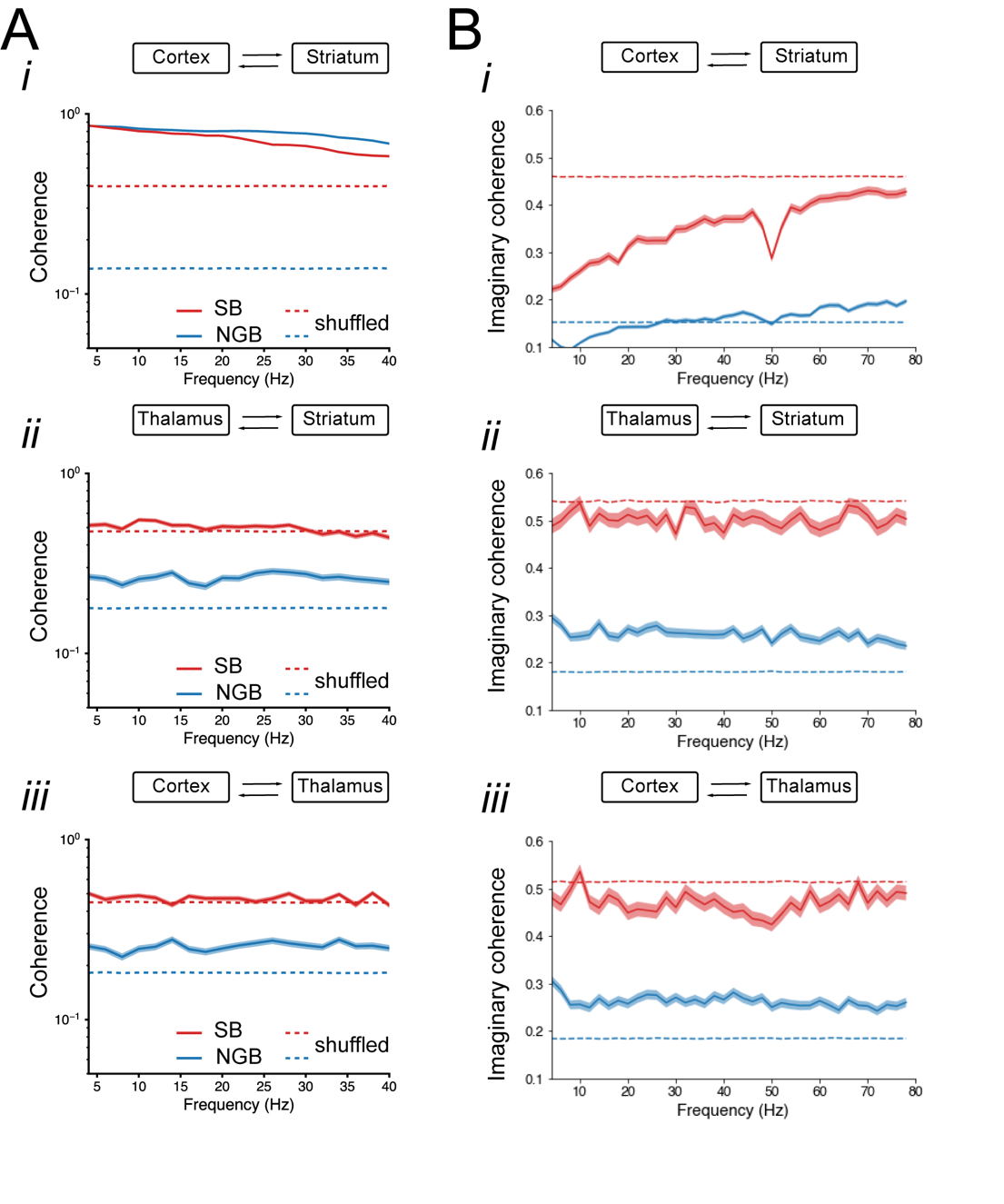
**

**Supplemental Figure 9: Real and imaginary cross-spectral coherence for SB and NGB events.** (**A**) Coherence analysis in the 4-40Hz frequency bands for corticostriatal events (*i*), thalamostriatal events (*ii*) and corticothalamic events (*iii*) segregated into SB-SB and NGB-NGB events. Note the overall broad coherence across frequencies and the large coherence for both SB and NGB events for cortex-striatum, whereas coordinated activity between other brain regions seems to be mostly during NGB events. (**B**) Coherence analysis of the imaginary part was also performed on co-occurring corticostriatal (*i*), thalamostriatal (*ii*) and corticothalamic (*iii*) SB-SB and NGB-NGB events to reveal potential non-zero lag interactions. Overall, NGB events appear to be significantly coherent between brain regions, while SB events do not exceed coherence values that would be expected by chance assuming no coherence. Shuffled data (dashed lines) signifies the 95^th^ percentile of the shuffled (null) distribution. Individual bursts as detected in all animals are pooled according to type and brain region in A and B.

**
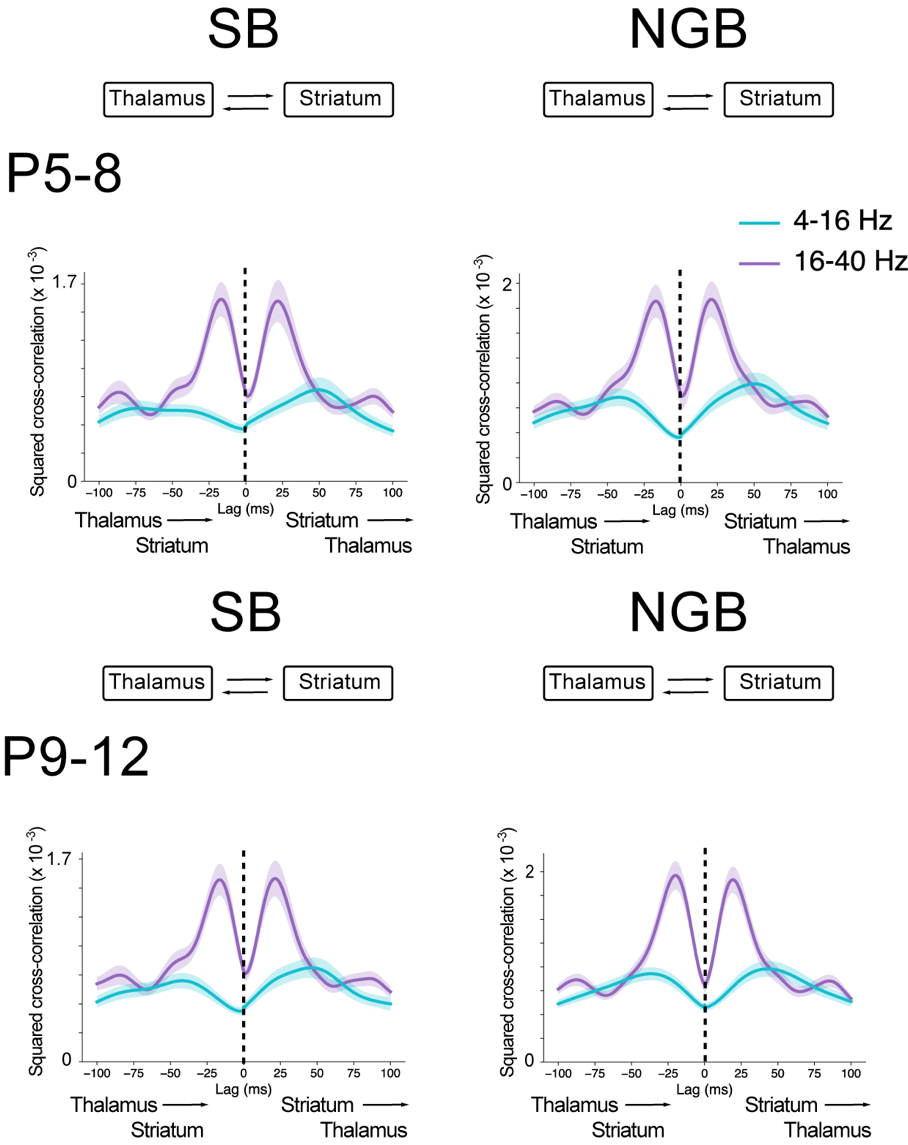
**

**Supplemental Figure 10: Cross-correlation analysis suggests minimal interaction between thalamus and striatum.** Results from cross-correlation analysis for SB and NGB events that occur between P5-8 (left) and between P9-12 (right) in thalamus and striatum. Note the overall consistently low values for the cross-correlation independent of age or type of burst. Individual bursts as detected in all animals were pooled according to type and age.

**
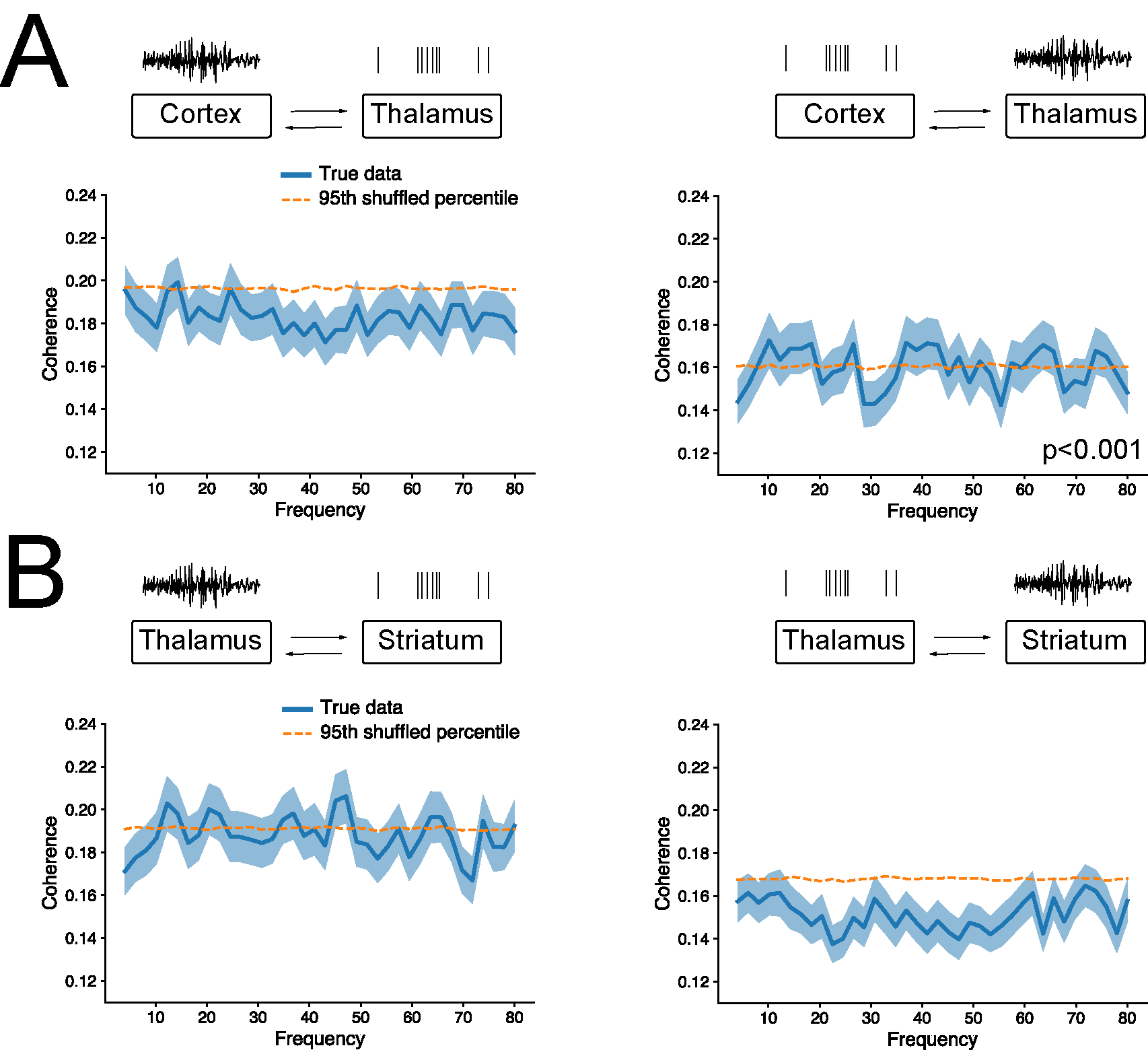
**

**Supplemental Figure 11: Spike-field coherence analysis between cortex-thalamus and thalamus-striatum.** (**A**) Spike-field coherence for cortico-thalamic interactions. Spike-field coherence for cortico-striatal interactions. Although no significant interaction was observed for cortex LFP with spiking in the thalamus (left, p<0.431) we find a significant interaction of the cortical spiking with thalamic LFP (right, p<0.001). (**B**) Spike-field coherence for striatal-thalamic interactions. No significant interaction was observed for thalamic LFP with spiking in the striatum (left, p<0.431) but a trend was seen for an interaction between thalamic spiking with the striatal LFP (right, p<0.084). Dashed lines indicate 95^th^ percentile range based on null distribution (shuffled data). Individual bursts and burst-associated MUA as detected in all animals were pooled according to brain region.

**
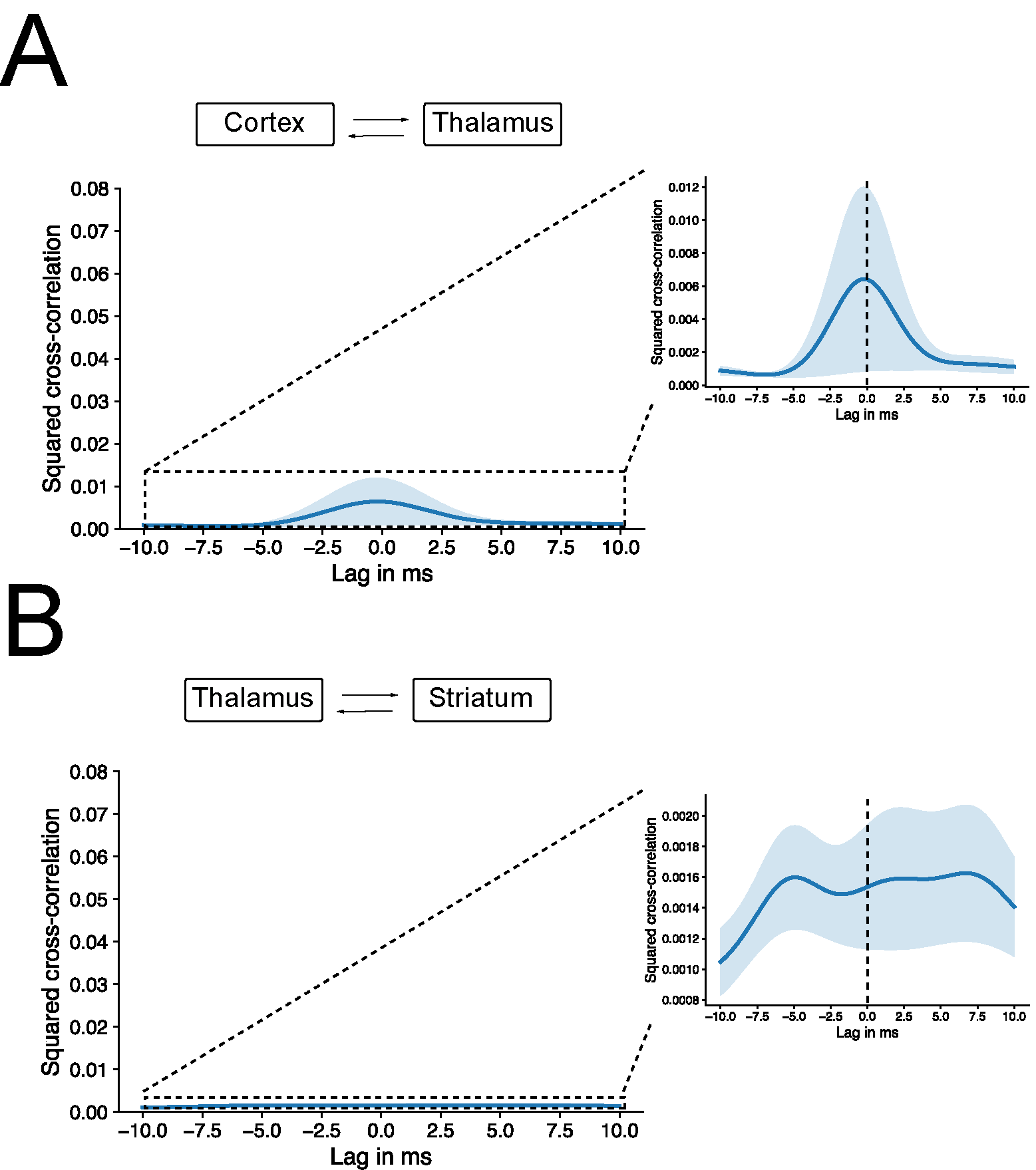
**

**Supplemental Figure 12: Spike-train cross-correlation analysis between cortex-thalamus and thalamus-striatum.** (**A**) Spike train cross-correlation between cortex and thalamus does not reveal evidence for one brain region driving other more strongly (peak of mean = -0.22 ms, one-sample *t*-test, p = 0.49). (**B**) Spike train cross-correlation between thalamus and striatum does not reveal evidence for one brain region driving other more strongly (one-sample *t*-test, p = 0.80). Individual bursts as detected in all animals were pooled according to brain region.

**
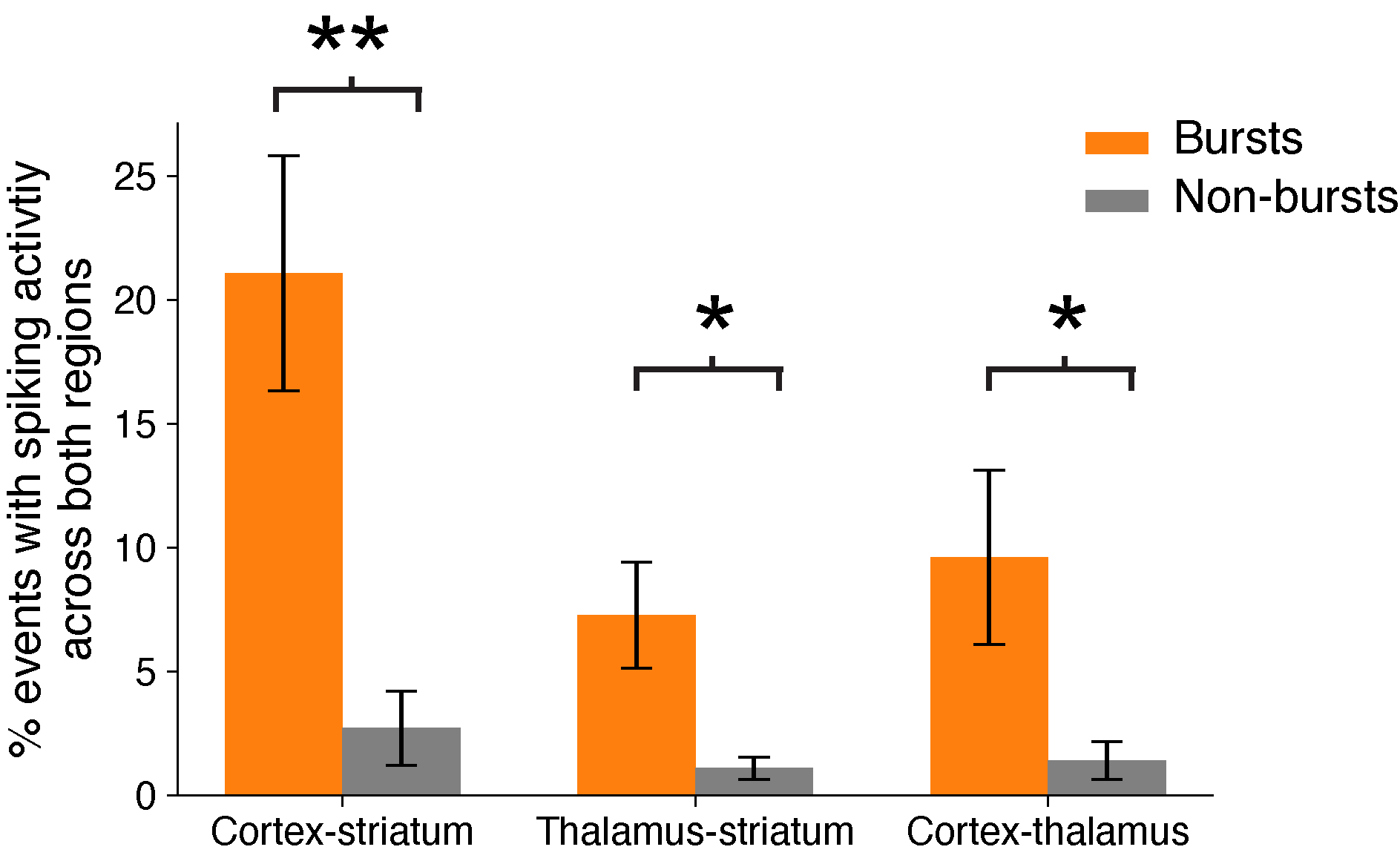
**

**Supplemental Figure 13: Mutual increases in spiking activity during co-occurring bursts are most pronounced between cortex and striatum.** Barplots of the number of co-occurring bursts that both exhibited an increase in spiking activity as a proportion of those where only one of the regions exhibited an increase in spiking activity. This was seen for all three pairs of brain regions but was most pronounced for cortex – striatum (cortex-striatum: Welch's t-test t(21)=3.44, p=0.002; thalamus-striatum: Welch's t-test t(15)=2.71, p=0.016; cortex-thalamus: Welch's t-test t(15)=2.18, p=0.046; cortex-striatum vs thalamus-striatum: Welch’s t-test t(23)=2.52, p=0.019; cortex-striatum vs cortex-thalamus: Welch’s t-test t(29)=1.85, p=0.074).

**
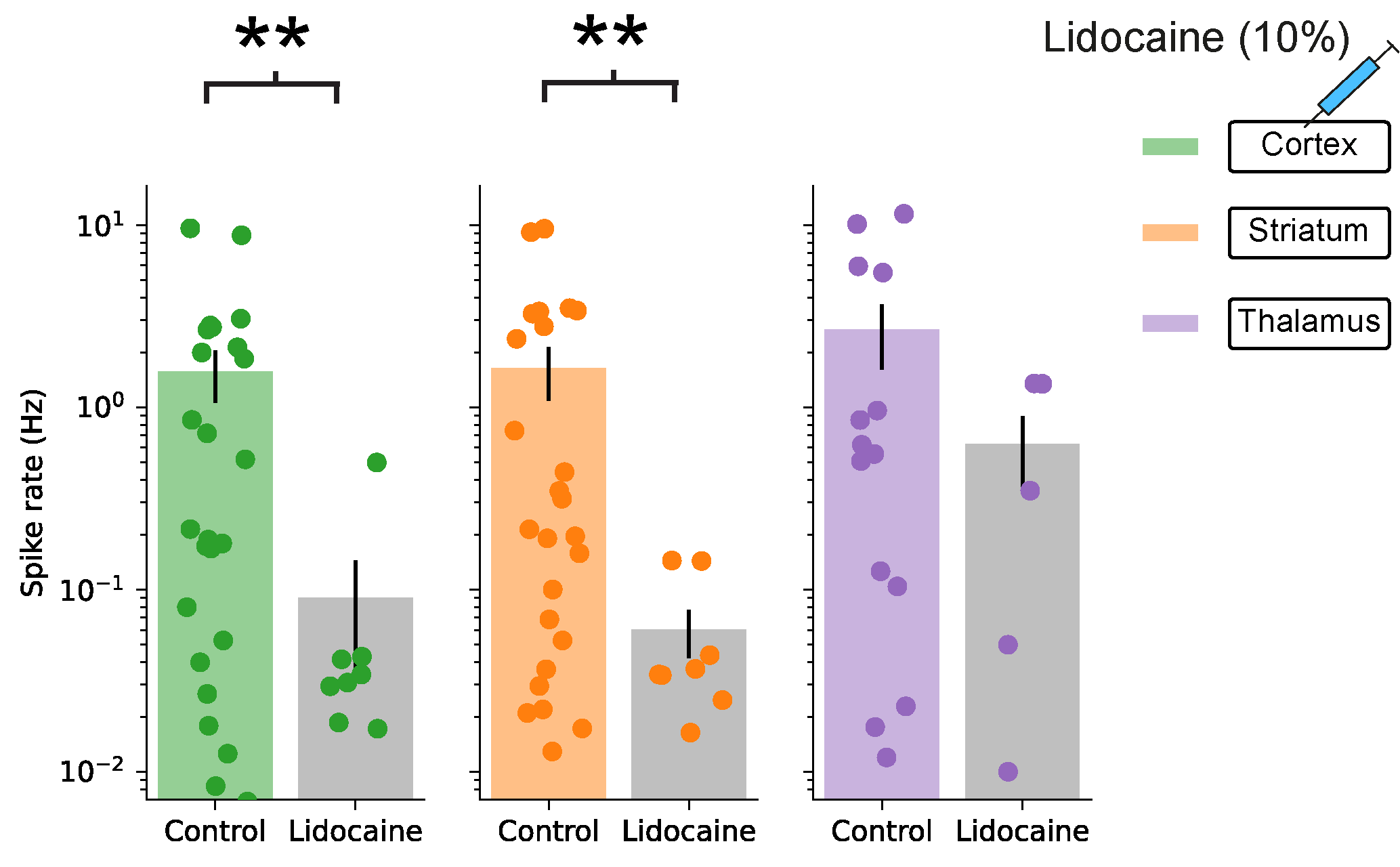
**

**Supplemental Figure 14: Cortical lidocaine injections significantly reduce the spike rate in cortex and striatum but not thalamus.** Barplots of the spike rates within detected bursts in cortex, striatum and thalamus after local lidocaine injections in cortex. Note the significant reduction in locally generated spike activity within striatum after inhibition of activity in cortex (vs. control cortex; t(25)=2.87, p=0.008, striatum; t(24)=2.92, p=0.008, thalamus; t(15)=1.82, p=0.089, Welch’s t-test, n = x control and n = 7 lidocaine).

**
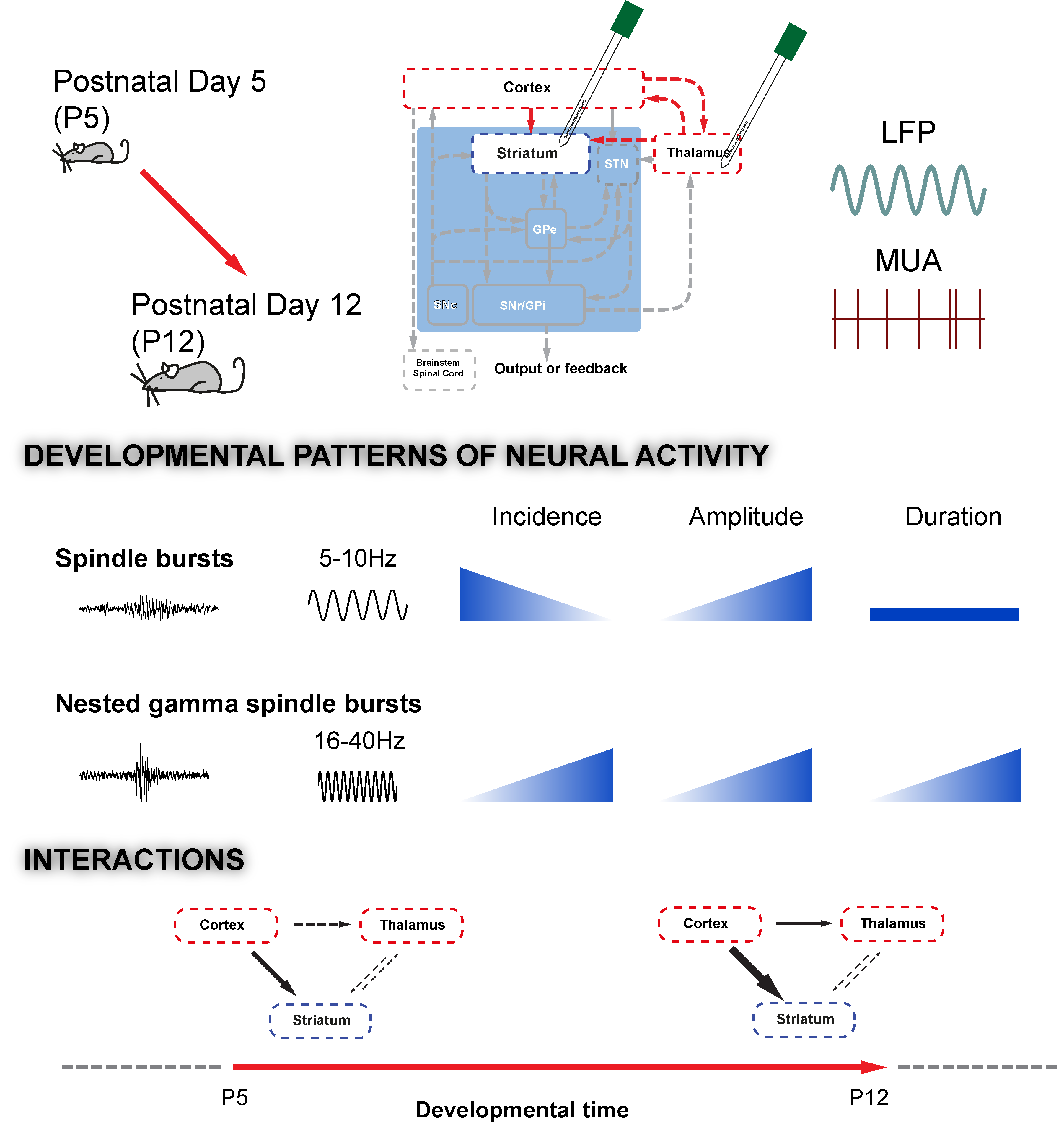
**

**Supplemental Figure 15: Summary diagram.**

**Supplemental Tables**

**Supplemental Table 1: Parameters for cortical burst events**

|  | **P5-6** | | **P7-8** | | **P9-10** | | **P11-12** | |
| --- | --- | --- | --- | --- | --- | --- | --- | --- |
|  | **NGB mean (SEM)** | **SB mean (SEM)** | **NGB mean (SEM)** | **SB mean (SEM)** | **NGB mean (SEM)** | **SB**  **mean (SEM)** | **NGB mean (SEM)** | **SB**  **mean (SEM)** |
| **Incidence (bursts per minute)** | 0.6 (0.29) | 4.6 (1.05) | 0.8  (0.4) | 4.6 (1.31) | 2.8  (0.78) | 4.0  (0.91) | 4.9  (0.6) | 2.4  (1.29) |
| **Amplitude (mV)** | 0.99 (0.20) | 0.356 (0.019) | 0.85 (0.064) | 0.39 (0.016) | 1.04 (0.075) | 0.53 (0.042) | 1.28  (0.13) | 0.73 (0.024) |
| **Duration (s)** | 3.1 (0.7) | 0.9 (0.04) | 3.2 (0.46) | 1.3 (0.04) | 5.4  (0.45) | 1.6 (0.08) | 5.1 (0.59) | 1.5  (0.09) |
| **Theta-alpha power** | 0.3 (0.03) | 0.3 (0.01) | 0.3 (0.03) | 0.3 (0.01) | 0.3  (0.03) | 0.3 (0.01) | 0.3 (0.01) | 0.2  (0.01) |
| **Theta-alpha peak frequency** | 12.1 (0.23) | 9.7 (0.31) | 12.2 (1.01) | 9.6 (0.71) | 8.3  (0.66) | 8.5 (0.37) | 9.3 (0.26) | 8.3  (0.33) |
| **Beta-low gamma power** | 0.5 (0.05) | 0.3 (0.01) | 0.5 (0.02) | 0.3 (0.02) | 0.4  (0.01) | 0.3 (0.01) | 0.4 (0.02) | 0.3  (0.02) |
| **Beta-low gamma peak frequency** | 20.9 (1.83) | 22.4 (0.3) | 21.7 (0.71) | 22.5 (0.58) | 22.6  (1.0) | 22.9 (0.87) | 24.3 (0.76) | 25.5  (0.33) |

**Supplemental Table 2: Parameters for striatal burst events**

|  | **P5-6** | | **P7-8** | | **P9-10** | | **P11-12** | |
| --- | --- | --- | --- | --- | --- | --- | --- | --- |
|  | **NGB mean (SEM)** | **SB mean (SEM)** | **NGB mean (SEM)** | **SB mean (SEM)** | **NGB mean (SEM)** | **SB**  **mean (SEM)** | **NGB mean (SEM)** | **SB**  **mean (SEM)** |
| **Incidence (bursts per minute)** | 0.7 (0.35) | 5.6 (0.99) | 0.8 (0.23) | 4.3 (1.01) | 2.6 (0.72) | 4.1 (1.0) | 5.2 (0.43) | 1.7 (0.77) |
| **Amplitude (mV)** | 1.03 (0.21) | 0.34 (0.019) | 0.95 (0.048) | 0.44 (0.023) | 1.04 (0.099) | 0.53 (0.058) | 1.43 (0.15) | 0.74 (0.029) |
| **Duration (s)** | 2.5 (0.17) | 1.0 (0.04) | 4.0 (0.07) | 1.3 (0.05) | 5.4 (0.66) | 1.5 (0.06) | 5.6 (0.78) | 1.4 (0.05) |
| **Theta-alpha power** | 0.3 (0.01) | 0.3 (0.01) | 0.4 (0.01) | 0.3 (0.01) | 0.3 (0.03) | 0.3 (0.02) | 0.2 (0.02) | 0.2 (0.01) |
| **Theta-alpha peak frequency** | 11.2 (1.09) | 9.4 (0.35) | 10.8 (0.46) | 8.8 (0.38) | 9.2 (0.61) | 8.6 (0.31) | 9.4 (0.25) | 8.4 (0.42) |
| **Beta-low gamma power** | 0.5 (0.05) | 0.3 (0.01) | 0.4 (0.02) | 0.3 (0.02) | 0.4 (0.01) | 0.3 (0.01) | 0.4 (0.01) | 0.3 (0.01) |
| **Beta-low gamma peak frequency** | 22.3 (0.73) | 22.5 (0.28) | 19.1 (0.39) | 23.0 (0.31) | 22.6 (0.92) | 23.3 (0.74) | 25.4 (0.77) | 25.5 (0.42) |

**Supplemental Table 3: Parameters for thalamic burst events**

|  | **P5-6** | | **P7-8** | | **P9-10** | | **P11-12** | |
| --- | --- | --- | --- | --- | --- | --- | --- | --- |
|  | **NGB mean (SEM)** | **SB mean (SEM)** | **NGB mean (SEM)** | **SB mean (SEM)** | **NGB mean (SEM)** | **SB mean (SEM)** | **NGB mean (SEM)** | **SB mean (SEM)** |
| **Incidence (bursts per minute)** | 0.4 (0.25) | 2.2 (0.97) | 0.4 (0.02) | 1.8 (0.7) | 2.3 (0.75) | 4.0 (0.48) | 5.4 (1.06) | 2.3 (1.01) |
| **Amplitude (mV)** | 0.96 (0.17) | 0.45 (0.045) | 0.92 (0.14) | 0.41 (0.045) | 0.99 (0.057) | 0.49 (0.078) | 1.04 (0.034) | 0.62 (0.047) |
| **Duration (s)** | 1.2 (0.07) | 0.7 (0.12) | 2.9 (0.19) | 1.1 (0.07) | 4.5 (1.17) | 1.4  (0.1) | 4.6 (0.28) | 1.5 (0.09) |
| **Theta-alpha power** | 0.3 (0.05) | 0.3 (0.01) | 0.3 (0.01) | 0.3 (0.05) | 0.3 (0.02) | 0.3 (0.01) | 0.2 (0.03) | 0.2 (0.01) |
| **Theta-alpha peak frequency** | 13.0 (0.77) | 11.0 (0.46) | 13.0 (0.31) | 9.6 (0.46) | 11.0 (1.0) | 9.3 (0.28) | 11.5 (0.18) | 10.0 (0.29) |
| **Beta-low gamma power** | 0.5 (0.04) | 0.3 (0.01) | 0.5 (0.02) | 0.3 (0.01) | 0.4 (0.02) | 0.3 (0.02) | 0.4 (0.01) | 0.3 (0.01) |
| **Beta-low gamma peak frequency** | 20.2 (0.69) | 21.9 (0.43) | 19.4 (0.55) | 23.0 (0.71) | 21.5 (1.76) | 24.2 (0.74) | 23.6 (1.49) | 26.2 (0.83) |

**Supplemental Table 4: Statistics for cortical burst events**

|  | **NGB mean (SEM)** | **SB mean (SEM)** | **df** | **Welch’s t** | **p (uncorrected)** | **Cohen’s d** |
| --- | --- | --- | --- | --- | --- | --- |
| **Duration (s)** | 5.52 (0.0844) | 1.44 (0.0192) | 1.83e+03 | 47.2 | 9.08e-319 | 1.63 |
| **Max RMS (μV)** | 193 (1.48) | 56.1 (0.628) | 2.25e+03 | 85.3 | 0 | 2.89 |
| **Negative peak (μV)** | -420 (3.19) | -126 (1.33) | 2.23e+03 | -85.4 | 0 | -2.89 |
| **Flatness** | 0.213 (0.00203) | 0.538 (0.00398) | 3.25e+03 | -72.8 | 0 | -2.24 |
| **Max slope** | 130 (1.23) | 42.8 (0.377) | 1.97e+03 | 68 | 0 | 2.33 |
| **Beta/low-gamma power** | 0.428 (0.00164) | 0.324 (0.00181) | 3.88e+03 | 42.5 | 9.88e-324 | 1.35 |
| **Theta-alpha power** | 0.273 (0.00179) | 0.268 (0.00188) | 3.85e+03 | 1.92 | 0.0549 | 0.0613 |
| **Inter-trough-interval (s)** | 0.0662 (0.000181) | 0.0551 (0.000177) | 3.78e+03 | 43.9 | 0 | 1.41 |
| **Spikes s−1** | 5.21 (0.166) | 1.35 (0.0508) | 1.97e+03 | 22.2 | 8.58e-98 | 0.761 |

**Supplemental Table 5: Statistics for striatal burst events**

|  | **NGB mean (SEM)** | **SB mean (SEM)** | **df** | **Welch’s t** | **p (uncorrected)** | **Cohen’s d** |
| --- | --- | --- | --- | --- | --- | --- |
| **Duration (s)** | 5.61 (0.0907) | 1.31 (0.0189) | 1.77e+03 | 46.4 | 4.64e-308 | 1.61 |
| **Maximum RMS (µV)** | 190  (1.52) | 49.3  (0.56) | 2.08e+03 | 87.2 | 0 | 2.99 |
| **Negative peak (µV)** | -417  (3.4) | -112 (1.21) | 2.05e+03 | -84.4 | 0 | -2.9 |
| **Flatness** | 0.213 (0.00207) | 0.576 (0.00401) | 3.27e+03 | -80.3 | 0 | -2.47 |
| **Maximum slope** | 136  (1.42) | 41.8 (0.361) | 1.84e+03 | 64.1 | 0 | 2.22 |
| **Relative beta-low gamma power** | 0.424 (0.00159) | 0.306 (0.00171) | 3.85e+03 | 50.5 | 0 | 1.61 |
| **Relative theta-alpha power** | 0.268 (0.00227) | 0.264 (0.00193) | 3.51e+03 | 1.34 | 0.179 | 0.0438 |
| **Inter-trough-interval (s)** | 0.0643 (0.000179) | 0.0527 (0.000162) | 3.63e+03 | 48.4 | 0 | 1.57 |
| **Spikes *s*^−1^** | 9.75 (0.341) | 2.51 (0.116) | 2.01e+03 | 20.1 | 6.14e-82 | 0.69 |

**Supplemental Table 6: Statistics for thalamic burst events**

|  | **NGB mean (SEM)** | **SB mean (SEM)** | **df** | **Welch’s t** | **p (uncorrected)** | **Cohen’s d** |
| --- | --- | --- | --- | --- | --- | --- |
| **Duration (s)** | 4.85 (0.0835) | 1.29 (0.0233) | 1.54e+03 | 41 | 2.89e-249 | 1.59 |
| **Maximum RMS (µV)** | 165  (1.12) | 50.4  (0.71) | 2.27e+03 | 86.9 | 0 | 3.35 |
| **Negative peak (µV)** | -406  (3.7) | -115  (1.52) | 1.78e+03 | -72.8 | 0 | -2.81 |
| **Flatness** | 0.224 (0.00234) | 0.612 (0.00474) | 2.01e+03 | -73.5 | 0 | -2.81 |
| **Maximum slope** | 131  (1.3) | 44.7 (0.504) | 1.73e+03 | 61.6 | 0 | 2.38 |
| **Relative beta-low gamma power** | 0.396 (0.00194) | 0.315 (0.00227) | 2.67e+03 | 27.2 | 1.47e-143 | 1.04 |
| **Relative theta-alpha power** | 0.259 (0.0023) | 0.24 (0.00236) | 2.72e+03 | 5.48 | 4.61e-08 | 0.21 |
| **Inter-trough-interval (s)** | 0.0591 (0.000169) | 0.0505 (0.000183) | 2.71e+03 | 34.6 | 3.36e-217 | 1.33 |
| **Spikes *s*^−1^** | 4.86  (0.146) | 6.51 (0.243) | 2.26e+03 | -5.81 | 7.09e-09 | -0.222 |

**Supplemental Table 7: Statistics for striatum versus thalamic NGB events**

|  | **Striatum mean (SEM)** | **Thalamus mean (SEM)** | **df** | **Welch’s t** | **p (uncorrected)** | **Cohen’s d** |
| --- | --- | --- | --- | --- | --- | --- |
| **Duration (s)** | 5.61 (0.0907) | 4.85 (0.0835) | 2.97e+03 | 6.17 | 7.75e-10 | 0.226 |
| **Maximum RMS (µV)** | 190 (1.52) | 165 (1.12) | 2.85e+03 | 13.2 | 7.37e-39 | 0.479 |
| **Negative peak (µV)** | -417 (3.4) | -406 (3.7) | 2.87e+03 | -2.15 | 0.0313 | -0.0794 |
| **Flatness** | 0.213 (0.00207) | 0.224 (0.00234) | 2.82e+03 | -3.3 | 0.000983 | -0.122 |
| **Maximum slope** | 136 (1.42) | 131 (1.3) | 2.97e+03 | 2.61 | 0.00908 | 0.0955 |
| **Relative beta-low gamma power** | 0.424 (0.00159) | 0.396 (0.00194) | 2.72e+03 | 11.3 | 8.37e-29 | 0.418 |
| **Relative theta-alpha power** | 0.268 (0.00227) | 0.259 (0.0023) | 2.93e+03 | 3.01 | 0.00263 | 0.111 |
| **Inter-trough-interval (s)** | 0.0643 (0.000179) | 0.0591 (0.000169) | 2.96e+03 | 21.2 | 2.93e-93 | 0.778 |
| **Spikes *s*^−1^** | 9.75 (0.341) | 4.86 (0.146) | 2.19e+03 | 13.2 | 4.06e-38 | 0.467 |

**Supplemental Table 8: Statistics for striatum versus thalamic SB events**

|  | **Striatum mean (SEM)** | **Thalamus mean (SEM)** | **df** | **Welch’s t** | **p (uncorrected)** | **Cohen’s d** |
| --- | --- | --- | --- | --- | --- | --- |
| **Duration (s)** | 1.31 (0.0189) | 1.29 (0.0233) | 3e+03 | 0.736 | 0.462 | 0.0251 |
| **Max RMS (μV)** | 49.3 (0.56) | 50.4 (0.71) | 2.94e+03 | -1.21 | 0.225 | -0.0415 |
| **Negative peak (μV)** | -112 (1.21) | -115 (1.52) | 2.96e+03 | 1.53 | 0.125 | 0.0524 |
| **Flatness** | 0.576 (0.00401) | 0.612 (0.00474) | 3.1e+03 | -5.8 | 7.5e-09 | -0.196 |
| **Max slope** | 41.8 (0.361) | 44.7 (0.504) | 2.73e+03 | -4.67 | 3.18e-06 | -0.161 |
| **Beta/low-gamma power** | 0.306 (0.00171) | 0.315 (0.00227) | 2.84e+03 | -3.05 | 0.00234 | -0.105 |
| **Theta-alpha power** | 0.264 (0.00193) | 0.24 (0.00236) | 3.03e+03 | 7.8 | 8.3e-15 | 0.265 |
| **Inter-trough-interval (s)** | 0.0527 (0.000162) | 0.0505 (0.000183) | 3.19e+03 | 8.85 | 1.4e-18 | 0.298 |
| **Spikes *s*^−1^** | 2.51 (0.116) | 6.51 (0.243) | 2.02e+03 | -14.9 | 1.41e-47 | -0.536 |

**Supplemental Table 9: Statistics for cortical versus striatal NGB events**

|  | **Cortex mean (SEM)** | **Striatum mean (SEM)** | **df** | **Welch’s t** | **p (uncorrected)** | **Cohen’s d** |
| --- | --- | --- | --- | --- | --- | --- |
| **Duration (s)** | 5.52 (0.0844) | 5.61 (0.0907) | 3.27e+03 | -0.715 | 0.475 | -0.0249 |
| **Max RMS (μV)** | 193 (1.48) | 190 (1.52) | 3.28e+03 | 1.18 | 0.24 | 0.041 |
| **Negative peak (μV)** | -420 (3.19) | -417 (3.4) | 3.27e+03 | -0.649 | 0.516 | -0.0227 |
| **Flatness** | 0.213 (0.00203) | 0.213 (0.00207) | 3.29e+03 | -0.123 | 0.902 | -0.00428 |
| **Max slope** | 130 (1.23) | 136 (1.42) | 3.22e+03 | -2.8 | 0.00515 | -0.0977 |
| **Beta/low-gamma power** | 0.428 (0.00164) | 0.424 (0.00159) | 3.29e+03 | 1.51 | 0.131 | 0.0526 |
| **Theta-alpha power** | 0.273 (0.00179) | 0.268 (0.00227) | 3.1e+03 | 1.76 | 0.0783 | 0.0615 |
| **Inter-trough-interval (s)** | 0.0662 (0.000181) | 0.0643 (0.000179) | 3.29e+03 | 7.36 | 2.29e-13 | 0.257 |
| **Spikes *s*^−1^** | 5.21 (0.166) | 9.75 (0.341) | 2.37e+03 | -12 | 4.75e-32 | -0.418 |

**Supplemental Table 10: Statistics for cortical versus thalamic NGB events**

|  | **Cortical mean (SEM)** | **Thalamus mean (SEM)** | **df** | **Welch’s t** | **p (uncorrected)** | **Cohen’s d** |
| --- | --- | --- | --- | --- | --- | --- |
| **Duration (s)** | 5.52 (0.0844) | 4.85 (0.0835) | 2.96e+03 | 5.66 | 1.65e-08 | 0.207 |
| **Maximum RMS (µV)** | 193 (1.48) | 165 (1.12) | 2.91e+03 | 14.8 | 5.27e-48 | 0.535 |
| **Negative peak (µV)** | -420 (3.19) | -406 (3.7) | 2.81e+03 | -2.84 | 0.00458 | -0.105 |
| **Flatness** | 0.213 (0.00203) | 0.224 (0.00234) | 2.81e+03 | -3.44 | 0.000587 | -0.127 |
| **Maximum slope** | 130 (1.23) | 131 (1.3) | 2.91e+03 | -0.132 | 0.895 | -0.00485 |
| **Relative beta-low gamma power** | 0.428 (0.00164) | 0.396 (0.00194) | 2.78e+03 | 12.5 | 1.03e-34 | 0.46 |
| **Relative theta-alpha power** | 0.273 (0.00179) | 0.259 (0.0023) | 2.65e+03 | 5.09 | 3.82e-07 | 0.189 |
| **Inter-trough-interval (s)** | 0.0662 (0.000181) | 0.0591 (0.000169) | 2.98e+03 | 28.7 | 5.16e-160 | 1.05 |
| **Spikes *s*^−1^** | 5.21 (0.166) | 4.86 (0.146) | 2.99e+03 | 1.56 | 0.12 | 0.0566 |

**Supplemental Table 11: Statistics for cortical versus thalamic SB events**

|  | **Cortical mean (SEM)** | **Thalamus mean (SEM)** | **df** | **Welch’s t** | **p (uncorrected)** | **Cohen’s d** |
| --- | --- | --- | --- | --- | --- | --- |
| **Duration (s)** | 1.44 (0.0192) | 1.29 (0.0233) | 3.03e+03 | 4.72 | 2.47e-06 | 0.161 |
| **Maximum RMS (µV)** | 56.1 (0.628) | 50.4 (0.71) | 3.18e+03 | 6.02 | 1.96e-09 | 0.203 |
| **Negative peak (µV)** | -126 (1.33) | -115 (1.52) | 3.15e+03 | -5.14 | 2.92e-07 | -0.174 |
| **Flatness** | 0.538 (0.00398) | 0.612 (0.00474) | 3.08e+03 | -11.9 | 4.32e-32 | -0.405 |
| **Maximum slope** | 42.8 (0.377) | 44.7 (0.504) | 2.81e+03 | -3.04 | 0.00242 | -0.104 |
| **Relative beta-low gamma power** | 0.324 (0.00181) | 0.315 (0.00227) | 2.95e+03 | 3.14 | 0.00171 | 0.107 |
| **Relative theta-alpha power** | 0.268 (0.00188) | 0.24 (0.00236) | 2.96e+03 | 9.27 | 3.49e-20 | 0.317 |
| **Inter-trough-interval (s)** | 0.0551 (0.000177) | 0.0505 (0.000183) | 3.37e+03 | 18.1 | 5.9e-70 | 0.604 |
| **Spikes *s*^−1^** | 1.35 (0.0508) | 6.51 (0.243) | 1.51e+03 | -20.8 | 7.98e-85 | -0.782 |

**Supplemental Table 12: Statistics for cortical versus striatal SB events**

|  | **Cortical mean (SEM)** | **Striatum mean (SEM)** | **df** | **Welch’s t** | **p (uncorrected)** | **Cohen’s d** |
| --- | --- | --- | --- | --- | --- | --- |
| **Duration (s)** | 1.44 (0.0192) | 1.31 (0.0189) | 4.48e+03 | 4.47 | 8.03e-06 | 0.134 |
| **Maximum RMS (µV)** | 56.1 (0.628) | 49.3 (0.56) | 4.42e+03 | 8.08 | 7.97e-16 | 0.242 |
| **Negative peak (µV)** | -126 (1.33) | -112 (1.21) | 4.44e+03 | -7.44 | 1.19e-13 | -0.222 |
| **Flatness** | 0.538 (0.00398) | 0.576 (0.00401) | 4.48e+03 | -6.69 | 2.52e-11 | -0.2 |
| **Maximum slope** | 42.8 (0.377) | 41.8 (0.361) | 4.47e+03 | 1.89 | 0.0589 | 0.0565 |
| **Relative beta-low gamma power** | 0.324 (0.00181) | 0.306 (0.00171) | 4.46e+03 | 7.14 | 1.05e-12 | 0.214 |
| **Relative theta-alpha power** | 0.268 (0.00188) | 0.264 (0.00193) | 4.47e+03 | 1.53 | 0.125 | 0.0458 |
| **Inter-trough-interval (s)** | 0.0551 (0.000177) | 0.0527 (0.000162) | 4.44e+03 | 10.2 | 4.32e-24 | 0.304 |
| **Spikes *s*^−1^** | 1.35 (0.0508) | 2.51 (0.116) | 3.07e+03 | -9.19 | 6.83e-20 | -0.275 |
